# Turning Detection During Gait: Algorithm Validation and Influence of Sensor Location and Turning Characteristics in the Classification of Parkinson’s Disease

**DOI:** 10.3390/s20185377

**Published:** 2020-09-19

**Authors:** Rana Zia Ur Rehman, Philipp Klocke, Sofia Hryniv, Brook Galna, Lynn Rochester, Silvia Del Din, Lisa Alcock

**Affiliations:** 1 Translational and Clinical Research Institute, Faculty of Medical Sciences, Newcastle University, Newcastle upon Tyne NE2 4HH, UK; Rana.zia-ur-Rehman@newcastle.ac.uk (R.Z.U.R.); Brook.Galna@newcastle.ac.uk (B.G.); Lynn.Rochester@newcastle.ac.uk (L.R.); Silvia.Del-Din@newcastle.ac.uk (S.D.D.); 2 Clinical Ageing Research Unit, Campus for Ageing and Vitality, Newcastle University, Newcastle upon Tyne NE4 5PL, UK; philipp-klocke@gmx.de (P.K.); sh959@cam.ac.uk (S.H.); 3 Faculty of Medicine, University of Southampton, Southampton SO17 1BJ, UK; 4 Department of Psychology, University of Cambridge, Cambridge CB2 3EB, UK; 5 School of Biomedical, Nutritional and Sport Sciences, Faculty of Medical Sciences, Newcastle University, Newcastle upon Tyne NE2 4HH, UK; 6 The Newcastle upon Tyne NHS Foundation Trust, Newcastle upon Tyne NE1 1AA, UK

**Keywords:** inertial measurement unit (IMU), wearables, upper body, lower body, spatial-temporal characteristics, signal-based characteristics, validation, machine learning, PLS-DA

## Abstract

Parkinson’s disease (PD) is a common neurodegenerative disorder resulting in a range of mobility deficits affecting gait, balance and turning. In this paper, we present: (i) the development and validation of an algorithm to detect turns during gait; (ii) a method to extract turn characteristics; and (iii) the classification of PD using turn characteristics. Thirty-seven people with PD and 56 controls performed 180-degree turns during an intermittent walking task. Inertial measurement units were attached to the head, neck, lower back and ankles. A turning detection algorithm was developed and validated by two raters using video data. Spatiotemporal and signal-based characteristics were extracted and used for PD classification. There was excellent absolute agreement between the rater and the algorithm for identifying turn start and end (ICC ≥ 0.99). Classification modeling (partial least square discriminant analysis (PLS-DA)) gave the best accuracy of 97.85% when trained on upper body and ankle data. Balanced sensitivity (97%) and specificity (96.43%) were achieved using turning characteristics from the neck, lower back and ankles. Turning characteristics, in particular angular velocity, duration, number of steps, jerk and root mean square distinguished mild-moderate PD from controls accurately and warrant future examination as a marker of mobility impairment and fall risk in PD.

## 1. Introduction

Parkinson’s disease (PD) affects 145,000 people across the UK [1] and more than six million people worldwide, with prevalence expected to rise [2]. Movement impairment is a cardinal feature of PD, with motor symptoms, including slowness of movement (bradykinesia), reduced amplitude of movement (hypokinesia), stiffness and rigidity (particularly axial/trunk rigidity), stooped posture (excessive trunk flexion), reduced arm swing and resting tremor [3], which progress with disease duration [4].

Navigating complex and unpredictable environments requires adaptations to stepping patterns and altering walking direction through turning. People with PD reportedly turn on average >60 times every hour [5]. Turning deficits in people with PD are evident for both the upper body (head and trunk) and lower body (legs) [6]. Upper body movements during turning are more rigid, with segments turning en bloc [6,7]. People with PD tend to turn more slowly with less angular rotation, meaning turning requires more steps [5,8]. People with PD are more likely to turn using multiple steps rather than a pivot turn on one leg [6]. Difficulty turning in people with PD is associated with clinical outcomes, such as falls, a fear of falling, disease severity, freezing of gait and cognitive impairment [9,10,11,12,13,14].

Instrumented assessments of movement provide objective, quantifiable outcomes that offer increased sensitivity when compared to clinical scales and functional assessments [15,16]. Evaluating turning is important in people with PD who often fall while turning and impaired performance during turning is related to an increased fall risk [17,18]. In particular, turning deficits in PD are more evident in habitual settings compared to constrained laboratory settings [19] and developing techniques for monitoring turning in the community are required to capture subtle deficits not quantifiable through clinical evaluation. Identifying turns using inertial measurement units (IMUs) has previously been achieved by either evaluating step-by-step/stride-by-stride angle [20,21,22] or using IMU signal characteristics such as angular displacement and velocity [13,23,24,25,26]. An IMU on the lower back most closely reflects the movement of the center of mass and is commonly used for turn detection [24,25,26]. Alternative methods have been used for detecting turns, such as peak detection [24], angular displacement thresholds [25] and zero-crossing [13,23], which has often been used in PD studies [19,23]. People with PD often demonstrate axial rigidity, rotating their upper body prior to their lower body when turning. As such, the turn may be composed of many smaller turns and appropriate signal processing techniques are required to avoid underestimation of turn rotation magnitude. To fully understand and describe turning strategies, it is important to develop valid algorithms, which robustly detect the start and end of turns, and novel methods for using data acquired from various body segments.

Previous work has shown that turning characteristics, such as turn velocity, angle and jerk extracted from the lower back, are useful for PD classification [27,28]. People with PD display a lack of dissociation, i.e., a lack of segment coordination, in the rotation of head, pelvis, and feet (turning en bloc) [29]. This, in combination with slower movements, has been identified as common turning deficits in PD [6]. Quantifying these characteristics using multiple IMUs may improve the classification of PD. Exploiting turning characteristics from an array of IMUs attached to both the upper and lower body may represent a useful clinical marker of mobility.

A variety of measurement outcomes may be extracted from IMUs, including those that are signal-based (i.e., root mean square (RMS) and jerk) or spatiotemporal (i.e., turn duration, angle, direction). Evaluating movement throughout the various phases of a turn (start, mid, and end phases) is important for enhancing our understanding of turning deficits in PD, but increases the number and dimension of the extracted features. Partial least square with discriminant analysis can overcome these issues, and may be an appropriate choice under such circumstances [30].

Given the complexity of turning, measuring turning performance may be useful for the classification of PD. Identifying measurement outcomes that are sensitive to pathology and predict clinical endpoints is important for classification models. Therefore, the aims of this study were to: (i) optimize and validate a custom algorithm for detecting turns and phases within the turn; (ii) extract a range of turning characteristics using multiple IMUs worn on the upper and lower body; and (iii) investigate the contribution of turning characteristics and sensor location in the classification of PD. We hypothesized that characteristics associated with movement speed and smoothness and the number of steps would be important in the classification of PD. We anticipated that characteristics extracted from the upper and lower body segments would both be influential.

## 2. Materials and Methods

### 2.1. Participants

ICICLE-GAIT is a collaborative study with ICICLE-PD, an incident cohort study (Incidence of Cognitive Impairment in Cohorts with Longitudinal Evaluation—Parkinson’s disease) conducted between June 2009 and December 2011 [31]. Participants were recruited as part of the “Incidence of Cognitive Impairment in Cohorts with Longitudinal Evaluation—GAIT (ICICLE-GAIT) study” [31]. PD participants were included provided they had a diagnosis of idiopathic PD according to the UK Brain Bank criteria [32]. PD participants were excluded if they had Parkinsonism disorders (i.e., drug-induced Parkinsonism, vascular Parkinsonism), atypical forms of Parkinson’s (i.e., Progressive Supranuclear Palsy, Multiple System Atrophy), significant cognitive impairment (MMSE score <24) or insufficient knowledge of working English. A group of controls of similar age and sex were recruited from the local community providing they were aged >60 years, able to walk independently and had no significant cognitive impairment, mood or motor disorder. Ethical approval was granted from the local NHS Research Ethics Committee: (REC: 09/H0906/82). Participants provided written informed consent in accordance with the Declaration of Helsinki [33]. The data presented here were part of a longitudinal study and represent data collected at the 36 month assessment. In total, 93 participants were included: 37 people with PD and 56 older adult controls.

### 2.2. Demographics and Clinical Measures

Demographics such as age, sex, height, mass, and body mass index (BMI) were obtained. Global cognition was assessed using the Mini-Mental State Examination (MMSE) [34] with higher scores indicating greater cognitive impairment. Balance confidence was measured with the Activity specific Balance Confidence scale (ABC) [35] with higher scores indicating reduced balance confidence.

Clinical measures were recorded for PD participants only. To assess the severity of PD motor symptoms, participants were classified according to the Hoehn and Yahr scale ranging from 0 (no symptoms) to 5 (wheelchair bound or bedridden unless aided) [36], as well as Part III of the modified Movement Disorder Society version of the Unified Parkinson’s Disease Rating Scale (MDS-UPDRS) ranging from 0 (no motor symptoms) to 132 (severe motor symptoms) [37]. The levodopa equivalent dose (LEDD mg/day) was calculated according to published criteria [38]. PD participants were assessed ON medication (approximately 1-h post dopaminergic medication). The new Freezing of Gait questionnaire (nFOG) was used to describe the proportion of PD participants who experienced freezing of gait (score ≥ 1) [39].

### 2.3. Testing Protocol and Equipment

**Figure 1:**
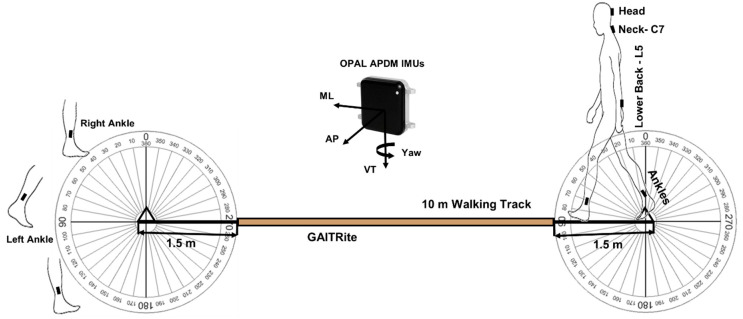
Testing protocol for detecting turning start and end during gait assessment.

As part of the gait assessment, participants completed four intermittent straight walks across an instrumented walkway. Participants initiated walking from behind a line of tape 1.5-metres from the walkway at a self-selected preferred pace until they passed another line of tape 1.5-metres beyond the end of the walkway [40]. Participants were instructed at the end of each walk to turn around (180 degrees) and wait for a command to walk back (Figure 1). Turn direction or turn strategy was not prompted or stipulated. A 2D color video camera (Logitech QuickCam 9000 Webcam, 30 Hz, resolution: 1600 × 1200 pixels) was positioned to cover the assessment area and was primarily used for data verification purposes.

Five IMUs containing a tri-axial gyroscope, accelerometer and magnetometer (APDM, Inc., Portland, OR, USA) were attached to the participants head, neck (cervical spine C7), lower back (lumbar spine L5) and ankles (lateral shank, superior to the medial malleoli), as shown in Figure 1. Adjustable velcro straps were used to attach IMUs at the head and ankles. IMUs attached to the neck and lower back were secured directly to the skin using double-sided adhesive and Hypafix (BSN Medical Limited, Hull, UK). Data were streamed and recorded synchronously from all five sensors sampling at 128 Hz (accelerometer range: ±6 g, gyroscope range: ±2000 degree per second, magnetometer range: ±6 Gauss). The intended purpose of this protocol was to assess gait. As the IMU’s recorded the full walking condition (intermittent walks and 180 degree turns), it was possible to pursue secondary analysis of covert turning behaviors.

### 2.4. Turn Detection and Algorithm Development

For the purpose of this study, a turn was defined as a rotation of the IMU on the lower back ≥90 degrees about the vertical axis with a duration of 0.5–10 s. Gradual turns (defined as turns ≥10 degrees and ≤0.5 s in the same direction) were merged [23]. Using this definition, an algorithm was developed (Algorithm 1) and implemented in MATLAB^®^ (R2019a, Mathworks, Natick, MA, USA).

Turn orientation was estimated based on the integration of angular velocity about the vertical axis. Kalman Filtering (by fusing 3D acceleration, gyroscope, and magnetometer signals) was used to estimate Euler angles to extract the orientation for all IMUs. For the upper body (head, neck, and lower back), static gyroscopic biases (due to factors such as temperature) and noise were removed with a compensation algorithm. This type of algorithm was chosen to avoid drift due to the integration [41]. For the lower body (left and right ankle), Kalman Filtering was used to avoid incorrect estimation of the gyroscopic bias, due to faster movement of the feet. For this, the “ahrsfilter” function in MATLAB^®^ was used to fuse the accelerometer, gyroscope and magnetometer data to estimate turn orientation. To detect possible movement in the body segments (head, neck, lower back, and ankles) around the vertical axis, zero-crossing of the angular velocity in the vertical direction was used. Based on the detected fluctuations in terms of yaw angles, gradual turns were combined for the head, neck and lower back IMU. The number of steps were quantified from the ankle IMUs when a turn angle of ≥30 degrees was detected around vertical axes.

All turns ≥90 degrees detected from the lower back IMU (L5) were considered for analysis. A 30 degree threshold was used for the head, neck, and ankles based on the lower quartile of the overall turn angle magnitude. To avoid the effect of turn direction on the ankle data, we organized ankle data according to whether they were inner (right ankle for a right turn, left ankle for a left turn) or outer (right ankle for a left turn, left ankle for a right turn) with respect to the turn. The start of a turn (turn start) and the end (turn end) were identified for each IMU, based on the algorithm in Algorithm 1.
**Algorithm 1.** Pseudo code for turning start and end detection.   01: #Data Access   02: **for** each IMU attachment #head(HD), Neck(C7), L5, Ankles(LA,RA)   03:  store calibrated **ω, a, m**   04: **end for**   05**:** #Data Filtering: Noise & Drift Removal   06: Access **ω**   07: **Compensation Algorithm:** for HD, C7, L5   08: Access **ω, a, m**   09: **Kalman Filtering (KF) Algorithm:** for Ankles (LA,RA)   10: #Every possible body segment rotation around VT   11: Access VT of **ω**   12: **Zero-Crossing:** for HD, C7, L5, Ankles   13: #Orientation Estimation   14: **Integration** ω VT to get Yaw: for HD, C7, L5   15: **Sensor fusion** with KF to Euler to Yaw: for Ankles   16: #Final Turn Detection and Estimation for HD, C7, L5   17: Yaw angle (**ᴪ**) extraction based on zero-crossing   18: **for** every **ᴪ**   19**:**   #Gradual Turns Combination   20:  if **ᴪ > 10°** and for next consecutive turns if **intra turn**   21:   **duration < 0.5 s** and **ᴪ > 10°** and **same Direction**   22**:**    Combine these gradual turns   23:  end   24:  #Turns from HD & C7   25:  if **turn angle (θ) ≥ 30°** and **0.5 s ≤ turn duration < 10 s**   26**:**    Save turn start and end   27:    Save final gradual turn magnitude vector    28:  end   29:  #Turns from L5   30:  if **turn angle (θ) ≥ 90°** and **0.5 s ≤ turn duration < 10 s**   31**:**    Save turn start and end   32:    Save final gradual turn magnitude vector    33:  end   34: **end for**   35**:** #Turn Transition and Estimation from Ankles (LA, RA)   36: **for** every turn from L5   37:  Access the turn start and turn end from L5   38:  Access the turn direction from L5   39:  #**Inner and outer turn** detection to overcome direction biases   40:  if L5 direction is right   41:    **Inner turn = RA**   42:    **Outer turn = LA**   43:  else (L5 direction is left)   44:    **Outer turn = RA**   45:    **Inner turn = LA**   46:  end   47: **end for**   48: #Final turn transition from Ankles   49: **for** angles from Inner and Outer turn   50:  if **inner turn angle ≥ 30°**   51**:**    save the turn start and turn end   52:    save the turn magnitude vector   53:  end   54:  if **outer turn angle ≥ 30°**   55**:**    save the turn start and turn end   56:    save the turn magnitude vector   57:   end   58: **end for** #Turning features extraction based on turn start and end time

### 2.5. Algorithm Validation (Aim 1)

Data from 26 participants (10 PD and 16 controls) who were not included in the validation analyses were used for algorithm development and optimization (participant demographics are provided in the supplementary Table S3). Orientation estimation using only the gyroscope was tested initially to optimize the compensation algorithm to remove signal noise and drift. Gradual turns were merged when they summed to a total angular displacement close to 180 degrees. The algorithm was optimized using different thresholds for the head, neck and ankles. The turn angle threshold was optimized, based on the distribution of the turn angles, to ensure all turns were included.

To validate the algorithm for detecting turn start and end from the lower back, we used the 2D video capture of the session. Videos for 67 participants (27 PD and 40 controls), who had at least three turns (201 turns in total) were reviewed by two independent raters. To be consistent across both groups (PD vs. CL), the first three turns were considered for validation. Raters were instructed to record the frame number of the turn start and turn end using predetermined definitions based on the following visible kinematic events. Turn start was defined as the frame at which the toe off occurs for the step which initiated a change in walking direction (i.e., the foot which first externally rotates in the direction of the turn). Turn end was defined as the frame at which heel strike occurs for the step, which concludes the change in walking direction. Both raters were blinded to which group participants were in.

The raters provided a number of additional annotations, including turn direction, and the presence and proximity of a researcher walking beside the participant. These outcomes were used for descriptive purposes only. We also asked the raters to nominate which group (PD or control) they believed the participant in the video belonged to. This information was compared to the results of the classification analysis (Section 2.7).

### 2.6. Feature Extraction (Aim 2)

Based on the turn detection protocol described in Section 2.4, we used the turn start and end times to extract 425 turning characteristics. The characteristics were categorized as spatiotemporal (i.e., turn time, angular velocity) and signal-based (i.e., RMS, jerk). The pseudo code for extracting these characteristics is provided in Algorithm 2. For the spatiotemporal characteristics, total turn time (seconds), turn angle (degrees), peak angular velocity (degrees/second) and peak angular frequency (radians/second) were extracted from each turn. Characteristics were calculated as an average over the start, mid and end of each turn. The number of turns and turn direction were also determined. For the signal-based characteristics [30]: RMS of acceleration (g); angular velocity (radians/second) from the gyroscope; RMS, maximum, minimum and range of jerk (g/sec); and RMS, maximum, minimum and range of angular acceleration (radians/second^2^) were computed. Signal-based turning characteristics were extracted from each IMU (accelerometer and gyroscope) in each axis (vertical; VT, anterior-posterior; AP, mediolateral; ML, resultant; R). The number of steps to complete a turn was extracted from the ankle IMUs. Feature extraction was performed in MATLAB^®^.

### 2.7. Classification Modeling and Importance of Turning Characteristics (Aim 3)

Partial least square with the discriminant analysis (PLS-DA) method was used for the classification of PD from controls [42,43]. The influential characteristics in the classification model were identified using the PLS-DA variable importance (VIP) score [44]. Characteristics with the 0.8 < VIP < 1.0 were considered moderately influential and scores > 1 highly influential. This method is capable of handling a large number of multi-collinear variables relative to the sample size [42]. In the PLS-DA, different combinations of turning characteristics were inserted, based on the type (signal, spatiotemporal, combined) and IMU (head, neck, lower back, ankles) to determine the best combination of turning characteristics for optimal PD classification. Model performance and number of components in the model were determined, based on the leave one out cross-validation. Model predictive quality (cumulated Q^2^ index) and the variance captured by the independent (R^2^X, i.e., turning characteristics) and dependent variables (R^2^Y, i.e., classification groups PD vs. control) were assessed. Increasing the number of model components can increase the captured variance while reducing the overall predictive performance leading to unreliable models. The number of components was selected based on the cumulated Q^2^ index, which assesses the global fitness of the model. The cumulated Q^2^ index for each component should be > 0, with values close to 1 considered important [30]. Classification modeling was performed in R using the plsDA package [45].
**Algorithm 2.** Pseudo code for turning features extraction.    01: #Detected Turns    02: Access turn start and end timings with turning angle magnitude    03: #**Spatiotemporal** turning characteristics    04: **for** total turns detected    05:  **Turn time** = mean of (turn end time − turn start time)    06:  **Turn angle** = mean of turning angle magnitude vector    07:  minimum, maximum, and variability in turn time and angle vector    08:  **#Full turn angular velocity**    09:  Angular velocity = Turn angle/Turn time    10:   #**Angular frequency (angular velocity)** in start, mid & end of turn    11:  selection of 0.1 s in the start, mid, end of within turn    12:  mean & variability in (max of angular frequency in overall turn)    13:  mean & variability in (mean of 0.1 s window in the start, mid, end)    14:  #**Direction** of turn    15:  **left** turn if angle magnitude is negative or **right** otherwise    16:  #**Number** of turn/transitions    17:  length of start and end time vector    18: **end for**    19: #**Signal based turning characteristics**    20**:** Accessing the **ω, a** in VT, AP & ML directions    21: **Detrend** the **ω, a**    22: **Data filtering**: 4^th^ order low-pass Butterworth filter at 20 Hz cut-off    23: Getting **resultant** magnitude of VT,AP & ML for **ω, a**    24**: for each** start, mid, and end of turn    25:  **RMS** for **ω, a** in VT, AP, ML & R #Turn overall, start, mid & end    26:  #**Jerk**: rate of change of **a**    27**:  RMS, max, min, range** of each turn jerk in VT, AP, ML & R #Turn     28:   overall, start, mid & End    29:  #**Angular acceleration**: rate of change of **ω**    30**:  RMS, max, min, range** of each turn angular acceleration in VT, AP,     31:   ML & R #Turn overall, start, mid & end    32: **end for** #classification modeling and statistical analysis

### 2.8. Statistical Analysis

For algorithm validation, the limit of agreement (LOA) expressed both as absolute values and as a percentage of the mean were used to assess agreement between the two raters and between the raters and algorithm, for the PD and control groups separately. Spearman’s rho assessed relative agreement while the intra-class correlation (ICC (2, 1)) assessed absolute agreement between raters and algorithm. Predefined acceptance ratings for ICC(2,1) were: excellent (>0.900), good (0.750–0.899), moderate (0.500–0.749) and poor (<0.500) [46,47]. RMS error (RMSE) was also calculated to quantify the performance of the model. Data are presented in scatter and Bland-Altman plots for visual inspection of agreement between the raters and algorithm.

For visualization of group differences, turning characteristics were converted to standardized Z-scores. To evaluate group differences, parametric (student *t*-test) and non-parametric (Mann-Whitney U-test) tests were performed on each turning characteristic depending on the distribution (Shapiro-Wilk test). Statistical analyses were performed in MATLAB^®^ and SPSS^®^. A *p*-value < 0.05 was considered statistically significant.

## 3. Results

The groups were well matched for age (52 to 89 years), height, mass, BMI and global cognition (MMSE) (Table 1; *p* > 0.05). Balance confidence (ABC scale) was significantly reduced in PD compared to controls (*p* < 0.001). PD participants were of mild-to-moderate disease stage (Hoehn and Yahr II-III), and LEDD was, on average, 536 mg/day. No freezing episodes were observed during the assessment.

### 3.1. Turning Algorithm Validation (Aim 1)

Data from a sub-sample were used within the validation analyses (27 PD, 40 control). The groups were well matched for age, height and mass, and had a mean UPDRSIII score of 40.4 ± 12.6.

**Figure A1:**
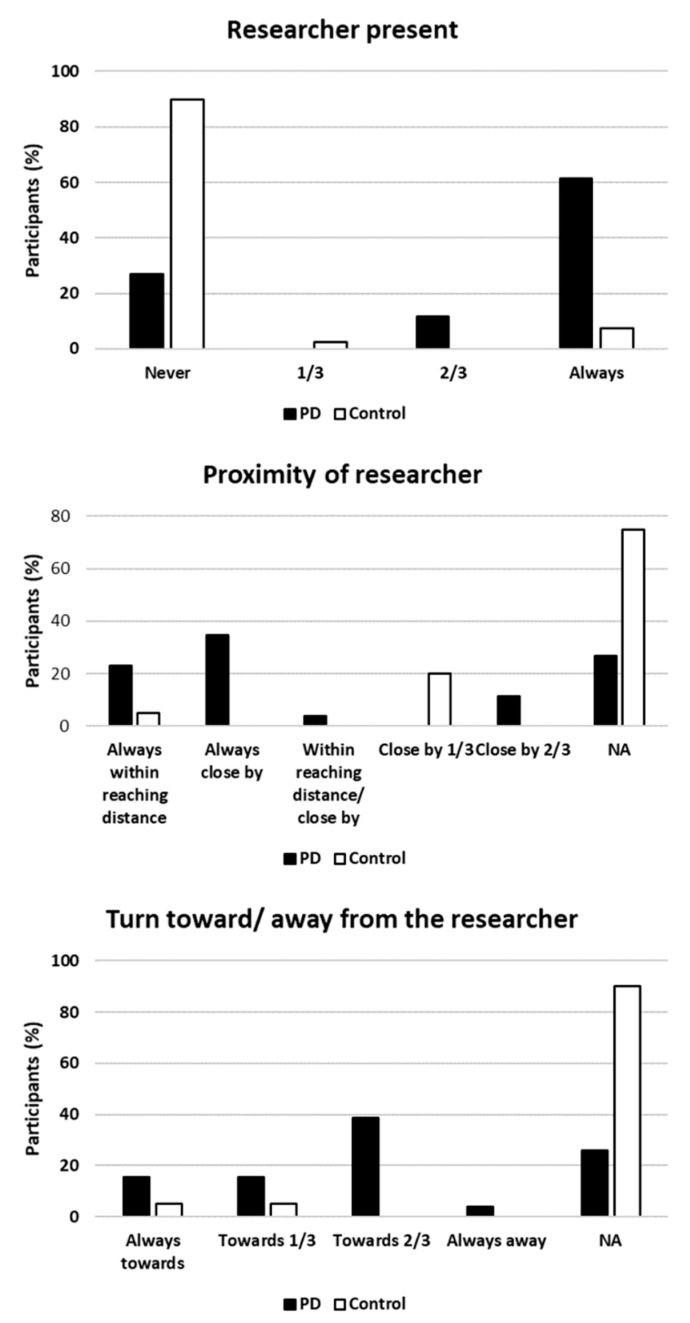
Bar charts presenting the proportion of PD and CL participants who completed the walking assessment with a researcher beside them, the proximity of the researcher and turn direction relative to the researcher (1/3 denotes 1 of 3 trials; 2/3 denotes 2 of 3 trials).

A researcher walked alongside 16 PD participants (62%) and 3 control participants (8%) for all walking trials. For 7 PD participants (27%) and 26 control participants (90%), they were unaccompanied during the walking trials. The remaining participants were accompanied on some, but not all, of the trials (1/3 or 2/3 trials). Of those participants for whom a researcher was present, the researcher either was close by or within reaching distance for 15 PD (58%) and 2 control (5%) participants for all walking trials. For a small number of participants (3 PD and 8 controls) a researcher was close by some of the time (1/3 or 2/3 trials). To understand whether the presence of a researcher influenced turn direction (right or left) the raters reported whether participants turned towards or away from the researcher. Four PD (15%) and 2 control participants always turned towards the researcher compared with 1 PD (4%) who always turned away. The majority of participants (14 PD and 2 control participants) for whom a researcher was present demonstrated no consistent turn direction with respect to the researcher (towards or away). Bar charts depicting these descriptors are provided in Appendix A Figure A1.

#### 3.1.1. Agreement between Raters

A good level of agreement was observed between the raters when identifying the turn start and end for PD and controls (Table 2, Scatter and Bland Altman plots are presented in Supplementary material Figure S1).

For the control group, both raters had excellent rho (0.99) and an ICC of 0.99 for turn start and turn end. RMSE and LOA were lower for turn start compared to turn end.

For the PD group, both raters had excellent rho (0.99) and an ICC > 0.98. RMSE was lower for turn start compared to turn end. LOA were lower for turn end compared to turn start.

#### 3.1.2. Agreement between the Raters and Algorithm

Table 2 shows the agreement between the raters and algorithm for identifying the turn start and end from the lower back (Bland Altman plots are presented in Supplementary Figure S1). Both raters had a consistent relative and absolute agreement for the control group for the turn start and end (0.99). RMSE and LOA were similar for Rater 1 and Rater 2 for controls with higher RMSE and LOA for turn end compared to turn start. Similarly, for the PD group, there was a perfect relative and absolute agreement between the raters and algorithm for both the turn start and turn end (0.99). Turn end had a higher RMSE and LOA as compared to turn start for both groups. Overall, greater RMSE was observed for controls compared to PD (0.6 s). RMSE and LOA varied between raters for the PD group. For both groups, a greater error was observed for the turn end compared to the turn start.

### 3.2. Extraction of Turning Characteristics (Aim 2)

**Figure 2:**
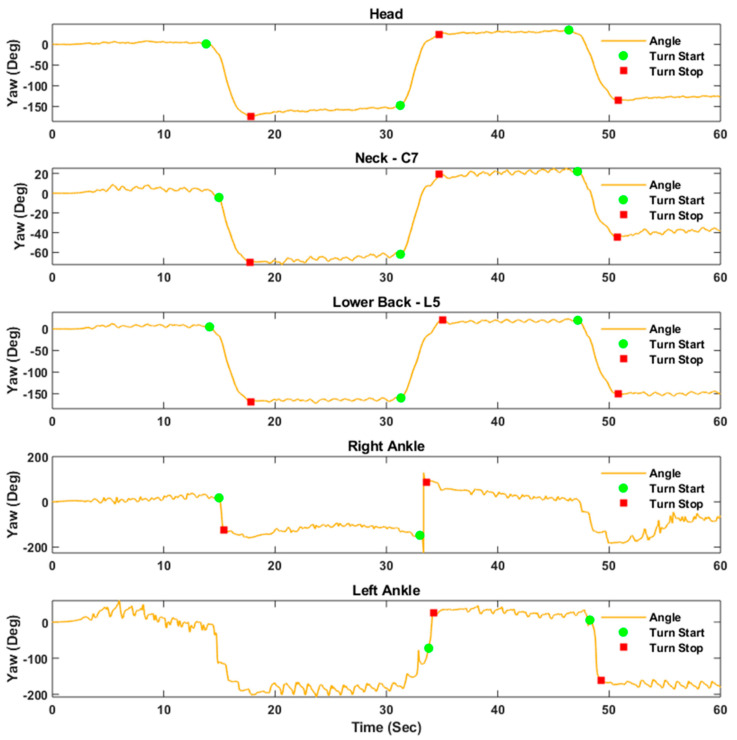
Turn start and turn end identification from each of the sensor attachments for one PD participant.

Figure 2 shows the estimated orientation of the IMUs at the head, neck, lower back, and ankles for a PD participant. This figure demonstrates that the vertical component of angular velocity may be used for orientation estimation after removing signal bias and noise using a compensation algorithm. At the ankles, orientation angles were estimated based on Kalman filtering. It is possible to see here that different turn strategies were employed. Based on the distribution of turn angle, a threshold of 30 degrees was used to quantify the number of transitions/steps from the ankle IMUs.

A total of 425 turning characteristics from the head, neck, lower back, and ankle IMUs were calculated. One-hundred-and-nine turning characteristics (21 spatiotemporal and 88 signal-based) were extracted from the head, neck, and lower back (total of 327). Forty-nine turning characteristics (9 spatiotemporal and 43 signal-based) were extracted from each ankle. Signal-based characteristics were extracted from each axis of the gyroscope and accelerometer (ML, AP, VT, R).

**Figure 3:**
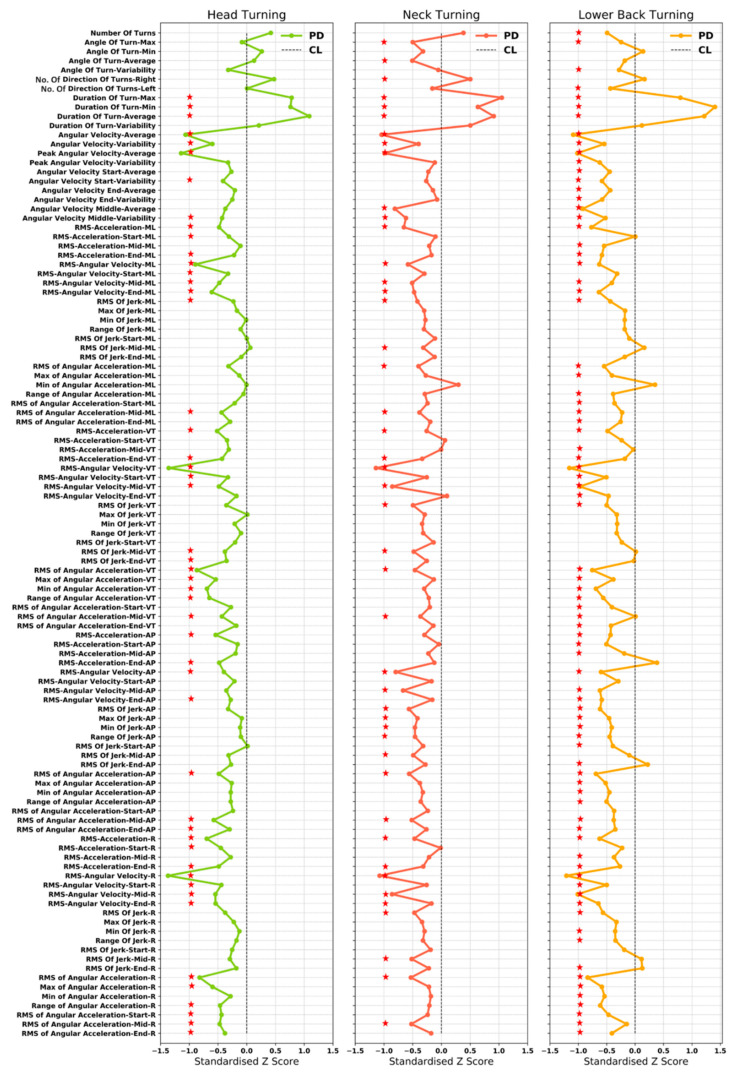
Group differences between turning characteristics extracted from the upper body for PD and CL (red star indicates significant group differences *p* < 0.05).

**Figure 4:**
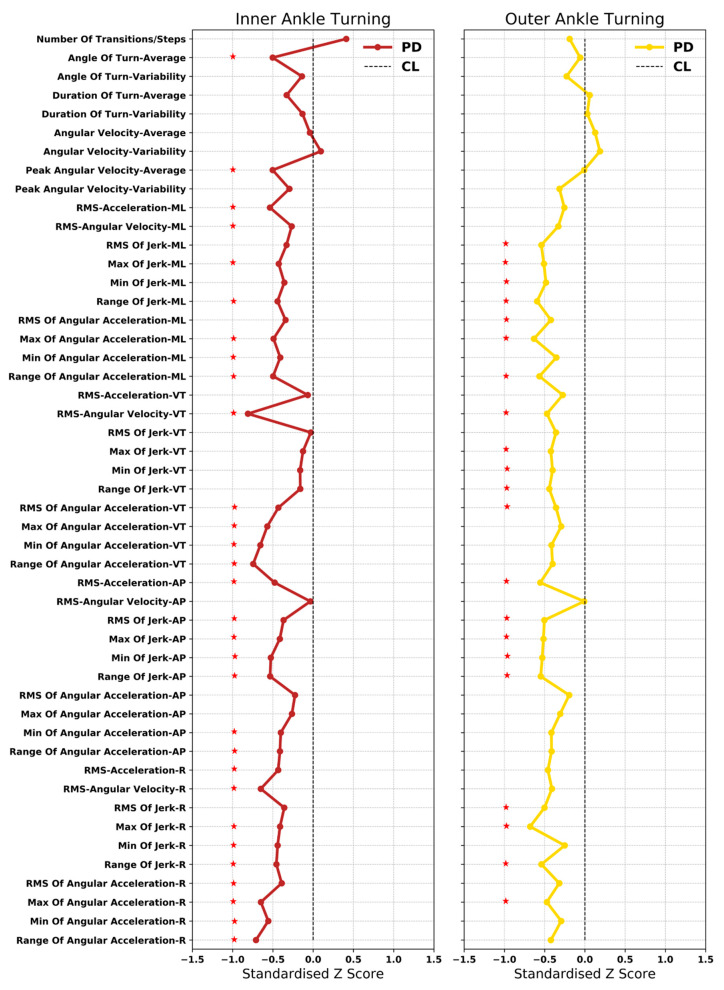
Turning characteristics extracted from the lower body for PD and CL (red star indicates significant group differences *p* < 0.05).

Figure 3 and Figure 4 show the difference between the PD and controls in terms of standardized Z-score for turning characteristics from the head, neck, lower back, and inner and outer ankle. Mean, standard deviation and *p* values for all characteristics are presented in Supplementary Table S1.

The following turning characteristics were found for each of the IMU locations: PD turned more frequently to the right compared to controls (CL); turn duration was longer; and angular velocity was reduced throughout the turn (overall, start, mid and end) in PD.

Turning characteristics from the head: PD participants had a more frequent head rotation higher compared to CL. From the signal-based turning characteristics, PD had lower RMS of acceleration and angular velocity overall, as well as during the start, mid, and end, compared to CL and this was true for all directions. PD participants had a lower RMS, and range of jerk in all directions. RMS of jerk at the start of the turn was slightly higher for PD (ML, AP and VT). RMS of jerk at the end of the turn was lower for PD compared to CL in all directions.

Similarly, RMS and range of angular acceleration were lower across the turn overall, as well as the start, mid and end in PD, in all directions.

Turning characteristics from the neck: Similar to the head, people with PD demonstrated more frequent and smaller neck (C7) rotations compared to CL. PD had lower RMS of acceleration and angular velocity compared to CL in all directions. Similarly, RMS of jerk and angular acceleration were lower for PD compared to CL in all directions.

Turning characteristics from the lower back: Fewer turns were detected for PD (according to defined criteria; Algorithm 1 Pseudo code) compared to CL. The turn angle was smaller (lower maximum and higher minimum) and less variable for PD compared to CL. Turn duration was more variable in PD compared to CL. RMS of acceleration and angular velocity were lower for PD compared to CL in all directions. RMS (minimum and maximum) and range of jerk were lower for PD in all directions. RMS of jerk was lower during the turn start and higher mid turn in all directions for PD. However, at the end of the turn, the RMS of jerk was lower in ML and VT directions and higher in the AP and R directions, for PD. PD also had lower RMS of angular acceleration in all directions.

Turning characteristics from the inner ankle: PD participants took more steps to complete the turn. The turn angle was smaller in PD compared to CL. Angular velocity was more variable in PD compared to CL RMS of acceleration, angular velocity, jerk, and angular acceleration were lower for PD compared to CL.

Turning characteristics from the outer ankle: PD participants took fewer steps with the outer ankle compared to CL. Almost the same turn angle and turn duration was observed in both groups; however, turn angle was less variable in PD compared to CL, with higher angular velocity observed in PD. RMS of acceleration, angular velocity, jerk and angular acceleration were lower for PD compared to CL.

### 3.3. Classification of PD (Aim 3)

**Figure 5:**
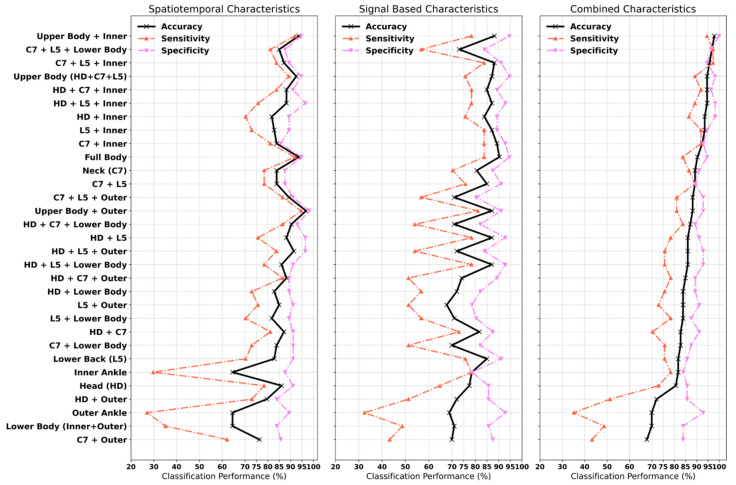
Classification performance of partial least square discriminant analysis (PLS-DA) trained on various combinations of IMU locations HD; head, C7; neck, L5; lower back, Inner; inner ankle, Outer; Outer ankle.

For each PLS-DA model, the explained variance captured by the independent (R^2^X) and dependent (R^2^Y) variables, overall model quality (cumulative Q^2^ index), and information about the number of selected components in the model is provided in Supplementary Table S2. Figure 5 shows the model performance trained for each sensor location separately and in combination with other sensor locations (classification results are detailed in Appendix A Table A1). When the independent raters used the 2D videos to classify PD and control participants, classification accuracy was 76% (Appendix A Table A2).

Overall, the PLS-DA model trained on the upper body (head, neck and lower back) and inner ankle resulted in optimal classification accuracy of 97.85% with a sensitivity of 95% and specificity of 100%. The model that provided balance in sensitivity (97.30%) and specificity (96.43%) included turning characteristics from the neck, lower back and lower body (inner and outer ankle) and achieved a classification accuracy of 96.77%. Using only the spatiotemporal characteristics, optimal classification was achieved when the model included the upper body (head, neck and lower back) and outer ankle resulting in a classification accuracy of 96.77% with 94.59% sensitivity and 98.21% specificity. Using only the signal-based characteristics, optimal classification was achieved when the PLS-DA was trained using all sensor locations (upper and lower body) with a classification accuracy of 90.32% with 84% sensitivity and 95% specificity.

Models considering only sensors on the upper body (head, neck and lower back) resulted in a classification accuracy of 94.62% with 89% sensitivity and 98% specificity. Considering only sensors on the lower body (inner and outer ankle) resulted in a classification accuracy of 70% with a sensitivity of 49% and specificity of 84%. Full body (upper and lower) spatiotemporal characteristics offered improved classification accuracy (94.64%) compared to signal-based characteristics (90.32%). Signal-based characteristics derived from the lower back resulted in a maximum accuracy of 84%; adding the inner ankle characteristics improved classification accuracy to 94% (92% sensitivity, 95% specificity); and adding the head and neck improved accuracy by a further 2%. Spatiotemporal characteristics from the upper body (head, neck and lower back) performed better than signal-based characteristics; however, optimal results were achieved when combined. In contrast, signal-based characteristics from the lower body (inner and outer ankle) performed better than spatiotemporal characteristics alone.

Using a combination of spatiotemporal and signal-based characteristics, and relying on a single sensor location, the neck IMU provided the best classification accuracy of 89.25% compared to the lower back (81.72%). Using only spatiotemporal characteristics, the neck and lower back resulted in a classification accuracy of 84% and 83%, respectively. Using only the signal-based characteristics, the lower back resulted in a classification accuracy of 85% compared to the neck (81%). In the majority of models, turning characteristics derived from the inner ankle improved the classification accuracy compared to the outer ankle, the inclusion of which reduced sensitivity.

### 3.4. Important Characteristics in the Classification Model

**Figure 6:**
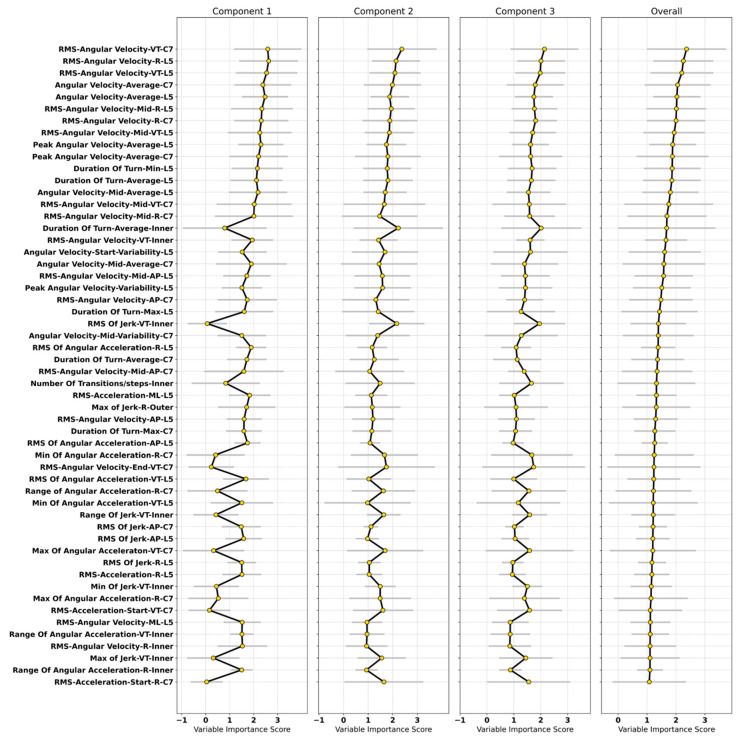
Important turning characteristics for PD classification based on the variable importance score from the PLS-DA. Each characteristic is arranged in ascending order according to the overall mean across all components.

The contribution and importance of individual turning characteristics in the classification models were quantified using the VIP in the PLS-DA (Figure 6). Characteristics were considered influential if they had VIP > 1.5 in any one of the PLS-DA components. Important turning characteristics for PD classification from a model (trained on the neck, lower back, inner and outer ankles) provided optimal balance in sensitivity and specificity.

Important spatiotemporal turning characteristics: Average angular velocity was an important spatiotemporal characteristic from the neck and lower back. From the lower back, turn duration (average, maximum, minimum); average angular velocity (mid turn); variability in angular velocity (turn start); and variability in the peak angular velocity were important. From the inner ankle, the number and duration of transitions/steps were influential. From the neck, angular velocity (average and variability; mid turn) and turn duration (average, maximum) were important.

Important signal-based turning characteristics: The most influential characteristics were the RMS of angular velocity from the neck (AP, VT, R) and lower back (all directions).

Similarly, in the middle of the turn, RMS of angular velocity for the lower back (AP, R) and the neck (AP, VT, R) were important. From the inner ankle, RMS of angular velocity (VT, R); RMS, minimum, maximum, and range of jerk in the VT direction; and range of angular acceleration (R) were important. From the lower back, RMS of angular acceleration (AP, VT, R); minimum angular acceleration (VT); RMS of acceleration (ML, R); and RMS of jerk (AP, R) were important. From the neck, angular acceleration (minimum and range; R, maximum; VT, R; and RMS of acceleration (turn start; VT and R directions; RMS of angular velocity (turn end; VT); RMS of jerk (AP) had greater importance. From the outer ankle, the maximum jerk (R) was important.

## 4. Discussion

In the present study, we have demonstrated that wearable technology can be used to detect turns whilst walking accurately in older adults and people with PD, and turning characteristics collected from a range of upper and lower body locations can also accurately distinguish between people with PD and age-matched controls. To the best of our knowledge, this is the first study to validate a turning algorithm for detecting the start and end of turns and quantify a comprehensive set of turning characteristics from a variety of sensor locations for PD classification. The combination of characteristics measured at the neck, lower back, and inner and outer ankles offered optimal sensitivity and specificity.

### 4.1. Turning Algorithm Validation

We found excellent agreement between the raters and the algorithm with higher error observed for the turn end for both groups. This was particularly true for controls, who turned faster (higher angular velocity). Turning speed may have influenced accuracy when identifying or detecting turn events. To validate the detection of the start and end of each turn, we defined kinematic events that could be detected through observation compared to the algorithm (which used velocity thresholds). However, the optimized algorithm was able to detect the small transitional rotation in the body accurately from the angular velocity, which is difficult to observe from the naked eye. Any small change in the body segment angle can be quantified, based on the zero crossing of angular velocity. Thus, using wearable IMUs is critical for the accurate and objective assessment of turning across a variety of body segments.

Previous studies [5,23] have used video annotations from independent raters to identify the turning phase, and verify the number of turns and turn duration while walking. Pham et al. [23] reported a mean absolute error for turn duration by considering the equal weights to error having different magnitudes, which may yield misleading optimistic results, as some turns can have error magnitude as compared to others. El-Gohary et al. [5] used a sample by sample approach from video rater annotations to quantify algorithm performance (sensitivity and specificity). Neither of the aforementioned studies validated the detection of turn start and end, which is particularly important given that turns may be initiated from standing still or walking and may end with movement termination or continuation of walking.

For comparison with published turning algorithms used with PD [5,23], Pham et al. [23], used six degrees of freedom (DOF) for orientation estimation using the gyroscope and acceleration signals from an IMU on the pelvis. El-Gohary et al. [5] used the quaternions obtained from an IMU on the pelvis to remove sensor drift and transform the gyroscopic data from body frame to the inertial frame. Both studies used a similar approach to orientation estimation. Instead, we took an alternative approach for accurate orientation estimation for different sensor locations, such as Kalman Filtering for the lower body and a compensation algorithm for the upper body to handle drift. The compensation algorithm was used to correct for signal noise and biases in gyroscopic data based on the sensor specification provided by the manufacturer. Integrating the vertical component of the gyroscope only [5] ensures it is computationally light weight and suitable for use in a variety of settings (laboratory and community). During the development phase, we observed that, for the ankles, the compensation algorithm could be unreliable due to the faster movement of the feet. Therefore, we used the full potential of the IMU, using all 9 DOF, to estimate the orientation with Kalman Filtering and estimate the biases accurately. The definitions used to extract the turn in both previous studies [5,23] was the same. Zero crossing of the vertical component of the gyroscope was used for identification of turn start and end. Pham et al. [23], found that a 90 degree threshold gave better results compared to the 45 degree threshold used by El-Gohary et al. [5]. Both studies [5,23] considered turns to have a duration of 0.5–10 s, while combining turns in the same direction with a duration <0.05 s according to El-Gohary et al. [5], and with duration <0.5 s and having turn magnitude >10 degrees according to Pham et al. [23]. During algorithm development, we took a number of additional steps: (1) Based on the distribution of the entire turn from the head and neck IMU, we observed turns that were undetected using a 90 degree threshold. To correctly identify individual turns of these segments, we selected a 30 degree threshold. (2) Turn direction was not stipulated, yet we observed that PD participants naturally chose to turn towards their right more frequently compared to controls who turned to the left. Further investigation revealed this finding was not related to the presence of a researcher. To overcome group differences in turn direction and investigate ankle function during turning, we introduced the concept of inner and outer ankles. As a result, we found the turn characteristics extracted from the inner ankle to have more discriminative power for classifying PD compared to the outer ankle.

### 4.2. Contribution of Turning in Classification of PD

#### 4.2.1. Classification of PD

This is the first study to investigate using turning characteristics from a combination of IMU locations for the classification of PD. Overall, classification with a single sensor ranged 70–89% accuracy, with 35–86% sensitivity and 84–92% specificity. The IMU on the neck gave optimal classification, followed by the lower back, inner ankle, head and outer ankle. The performance was further improved when turning characteristics were combined from other IMU locations; with a combination of the neck, lower back, head and inner ankle resulting in an optimal accuracy of 98% (sensitivity 95%, specificity 100%). Our findings are in line with previous work [27,28]. Using a sensor on the pelvis and ankles, Shah et al. [27] reported the AUC of 0.89 when using turn angle only (29 PD and 27 CL). Similarly, Hasegawa et al. [28] used eight sensors (attached at sternum, wrists, lumbar region, shank and feet) to quantify different domains of balance and reported an overall accuracy of 82.4% with a random forest classifier (144 PD and 79 CL). Of the gait and turning characteristics included in the random forest classifier, angular velocity was the most important in the classification of PD. We have previously used gait characteristics in the classification of PD [48,49], resulting in 73–93% accuracy. In addition, PLS-DA trained on the gait characteristics gave a classification accuracy of 70.42–88.73% (AUC: 78.4–94.5%) with a sensitivity of 72.84–90.12% and specificity of 60.3–86.89% [30]. The studies [27,28], which include both turning, gait and other characteristics confirm the value of including turning characteristics with gait characteristics for PD classification.

#### 4.2.2. Important Turning Characteristics

The turning characteristics which were most influential in the classification modeling were related to RMS of angular velocity (radians/second), angular velocity (turn angle/turn duration), turn duration, jerk, angular acceleration, number of steps/transitions per turn, and RMS of acceleration. The results from this study are in line with previous findings [28] confirming that turn velocity was the most important feature given by the random forest classifier. In another study [27], the variability of turn duration, jerk and turn angle gave better performance using logistic regression. Important signal-based characteristics identified in the present study, such as RMS of angular velocity, acceleration in the start, mid and end of the turn, were not included in previous work [27,28]. This RMS of angular velocity was the most influential signal-based characteristic in the present study when derived from the neck and lower back in the vertical direction.

Important turning characteristics reported in the present study are based on the model trained on characteristics derived from the neck (C7), lower back (L5) and lower body (inner and outer ankles), which resulted in optimal balance in sensitivity (97.30%) and specificity (96.43%) with an accuracy of 97%. In terms of body segment coordination during turning, older adults without PD sequentially rotate the head, followed by trunk and pelvis to turn [50,51,52]. Instead, people with PD exhibit the simultaneous movement of head and trunk, resulting in reduced head movement after the second step while turning [53]. In addition, due to en bloc movement of body segments in PD during turning, extracting turning characteristics from the start, mid and end of the turn were informative for PD classification. We found that the vertical and resultant components of RMS of acceleration from the neck at the start of the turn were important. Similarly, the vertical, anteroposterior and resultant components of the RMS of angular velocity (mid turn) and the vertical component at the end of turn were important from the neck. For the lower back (L5), the anteroposterior and resultant component of the RMS of angular velocity (mid turn) were important. Spatiotemporal characteristics extracted from the neck and lower back, such as angular velocity (mid turn), were also important. People with PD turn with a shorter step length [52], resulting in slower turning speeds, increased turn duration and a higher number of steps [54]. These differences in turning behaviors between PD and controls are reflective of hypokinesia [52].

Several turning characteristics from the inner ankle, such as turn duration, angular velocity, jerk, angular acceleration, the number of steps required to complete the turn were important. From the outer ankle, the only characteristic of importance was the maximum jerk. A reduction in segment rotation can influence stepping strategy during turning [55]. The number of steps becomes more prominent when the turning happens in a new direction [56], and a greater number of steps while turning may reflect increased task difficulty for older adults [57] and is associated with more severe PD symptoms [56]. An example of this is shown in Figure 2, when the PD participant required multiple steps to complete a turn, the number of which varied from one turn to the next.

### 4.3. Study Strengths, Limitations, and Directions for Future Work

Previous work has validated algorithms for turn detection based on video-derived annotations comparing the number of turns, turn phase and turn duration. In the present study, we validated an algorithm for detecting turns through identifying turn start and turn end. Developing robust means for detecting the start and end of a turn is important for subsequently derived turn characteristics. We selected thresholds based on the study setting; however, thresholds can be adjusted to capture a range of turn behaviors. Further work is required to determine appropriate thresholds for a variety of settings. Thresholds used in the lab may not be appropriate for use in the community where smaller turns may be required and thresholds may need to be adjusted for different sensor arrays and locations [5,12,13,19]. Investigating the impact of different thresholds is beyond the scope of this paper and will be considered in future studies along with the investigation of different turning strategies.

In this study, we present turn data using a semi-structured protocol during which turning behaviors were covertly assessed. The turn angular displacement was set (180 degrees) and initiation of each turn was prompted by passing a line on the floor. Turn area, turn direction (right or left) and turn strategy were not controlled; therefore, a range of turning behaviors were included in the present analysis. A variety of strategies may be used when turning (toward, lateral, pivot, incremental or delayed onset) [55]; however, this was not investigated.

A strength of the present study is the use of a multi-sensor array to quantify turning characteristics for PD classification. We have shown that turning characteristics from the neck, lower back, inner and outer ankles resulted in optimal balance in sensitivity and specificity. Previous studies attached the sensor on the lower back [23], pelvis and feet [5,13,19,27]. Further analyses may include exploring the temporal co-ordination between segments (sensor locations) which may be particularly important for PD [6,29]. Future work should investigate the interplay between turning performance and disease stage as well as other clinical outcomes, such as fall risk, cognitive decline and motor phenotype.

## 5. Conclusions

In this article, we demonstrated that turning during gait can be accurately identified with inertial measurement units, and turn characteristics from both the upper and lower body are important in the classification of PD. Turning characteristics extracted from the head, neck, lower back and inner ankle gave maximum accuracy of 97.85%, with the optimal balance in sensitivity (97.30%) and specificity (96.43%) achieved using turn characteristics from the neck, lower back, and inner and outer ankles. Characteristics related to movement speed (such as angular velocity), smoothness (such as jerk) and the number of steps taken to complete a turn were highly influential in the classification of PD. Further work is warranted to test the clinical utility of turning metrics captured in both laboratory and community-based settings.

## Figures and Tables

**Table 1:** Demographic and clinical outcomes for the PD and control group.

Demographics	CL (n = 56)	PD (n = 37)	*p*
Sex (Male/Female)	32/24	26/11	0.338
Age (years)	71.0 ± 7.1	70.1 ± 9.3	0.610
Height (m)	1.71 ± 0.08	1.68 ± 0.08	0.130
Mass (kg)	80.0 ± 12.9	77.2 ± 17.3	0.388
BMI (kg/m^2^)	27 ± 5	27 ± 6	0.799
MMSE (0–30)	29 ± 2	28 ± 2	0.137
ABCs (0–100)%	92 ± 11	77 ± 20	<0.001
Gait Speed (m/s)	1.12 ± 0.49	1.09 ± 0.36	0.724
LEDD, mg/day		534 ± 278	
Number of Freezers		7	
Disease Duration (years)		3.1 ± 0.2	
Hoehn and Yahr Stage (n)		HY II: 31	
		HY III: 6	
MDS-UPDRS III		41.1 ± 12.0	
		HY II: (39.6 ± 12.1)	
		HY III: (48.7 ± 8.5)	

ACRONYMS: BMI, Body mass index; MMSE, Mini-Mental State Examination; ABCs, Activity balance score; LEDD, Levodopa equivalent medical dosage; MDS-UPDRS, Movement disorders-Unified Parkinson’s disease rating scale.

**Table 2:** Statistics for validation of turning algorithm for control (n = 40) and PD (n = 27).

**Rater 1 vs. Rater 2**								
**Turn**	**Control**				**Parkinson’s Disease**			
	**RMSE**	**rho**	**ICC(2,1)**	**LOA**	**RMSE**	**rho**	**ICC(2,1)**	**LOA**
Start (s)	0.33	0.99	0.99	0.66 (4.6%)	0.42	0.98	0.99	0.83 (4.8%)
End (s)	0.44	0.99	0.99	0.87 (3.8%)	0.35	0.99	0.99	0.70 (3%)
**Rater 1 vs. Algorithm**								
**Turn**	**Control**				**Parkinson’s Disease**			
	**RMSE**	**rho**	**ICC(2,1)**	**LOA**	**RMSE**	**rho**	**ICC(2,1)**	**LOA**
Start (s)	0.50	0.99	0.99	0.97 (8.7%)	0.48	0.99	0.99	0.93 (5%)
End (s)	0.60	0.99	0.99	1.2 (6.7%)	0.59	0.99	0.99	1.2 (5.7%)
**Rater 2 vs. Algorithm**								
**Turn**	**Control**				**Parkinson’s Disease**			
	**RMSE**	**rho**	**ICC(2,1)**	**LOA**	**RMSE**	**rho**	**ICC(2,1)**	**LOA**
Start (s)	0.44	0.99	0.99	0.86 (8.8%)	0.39	0.99	0.99	0.77 (5.4%)
End (s)	0.60	0.99	0.99	1.2 (6.3%)	0.54	0.99	0.99	1.1 (6%)

ACRONYMS: RMSE, Root mean square error; rho, Spearman’s Rho correlation coefficient; ICC, Intra-Class correlation coefficient; LOA, Limit of agreement.

## Supplementary Materials

The following are available online at https://www.mdpi.com/1424-8220/20/18/5377/s1, Figure S1: Scatter plots and Bland Altman plots for validation of the turning start and turning end. Table S1: Statistical Analysis of Turning Characteristics from Lower Back (L5), Neck (C7), Head (HD), Inner Ankle (Inner), Outer Ankle (Outer). Table S2: Partial least square discriminant analysis (PLS-DA) model parameters trained on turning characteristics.

## Appendix A

**Table A1:** Classification accuracy (%) based on PLS-DA trained on a combination of characteristics.

Sensor Attachment	Spatiotemporal			Signal-Based			Combined		
	Specificity	Sensitivity	Accuracy	Specificity	Sensitivity	Accuracy	Specificity	Sensitivity	Accuracy
Head (HD)	91.07	78.38	86.02	85.71	64.86	77.42	85.71	72.97	80.65
Neck (C7)	87.50	78.38	83.87	87.50	70.27	80.65	91.07	86.49	89.25
Lower Back (L5)	91.07	70.27	82.80	91.07	75.68	84.95	85.71	75.68	81.72
HD + C7	91.07	81.08	87.10	87.50	72.97	81.72	91.07	70.27	82.80
HD + L5	96.43	75.68	88.17	92.86	78.38	87.10	91.07	78.38	86.02
C7 + L5	87.50	78.38	83.87	91.07	75.68	84.95	89.29	89.19	89.25
Upper Body	94.64	89.19	92.47	94.64	75.68	87.10	98.21	89.19	94.62
Inner Ankle	87.50	29.73	64.52	78.57	78.38	78.49	83.93	78.38	81.72
Outer Ankle	89.29	27.03	64.52	92.86	32.43	68.82	92.86	35.14	69.89
Lower Body	83.93	35.14	64.52	85.71	48.65	70.97	83.93	48.65	69.89
HD + Inner	89.29	70.27	81.72	89.29	75.68	83.87	98.21	86.49	93.55
HD + Outer	83.93	72.97	79.57	85.71	51.35	72.04	85.71	51.35	72.04
HD + Lower Body	89.29	72.97	82.80	82.14	56.76	72.04	89.29	75.68	83.87
C7 + Inner Ankle	85.71	81.08	83.87	92.86	83.78	89.25	92.86	91.89	92.47
C7 + Outer Ankle	85.71	62.16	76.34	87.50	43.24	69.89	83.93	43.24	67.74
C7 + Lower Body	91.07	72.97	83.87	82.14	51.35	69.89	87.50	75.68	82.80
L5 + Inner Ankle	89.29	72.97	82.80	89.29	83.78	87.10	94.64	91.89	93.55
L5 + Outer Ankle	91.07	75.68	84.95	78.57	51.35	67.74	91.07	72.97	83.87
L5 + Lower Body	89.29	70.27	81.72	80.36	56.76	70.97	87.50	78.38	83.87
HD + C7 + Inner Ankle	91.07	83.78	88.17	89.29	78.38	84.95	96.43	91.89	94.62
HD + C7 + Outer Ankle	89.29	86.49	88.17	89.29	51.35	74.19	89.29	78.38	84.95
HD + C7 + Lower Body	92.86	86.49	90.32	82.14	54.05	70.97	89.29	83.78	87.10
HD + L5 + Inner Ankle	96.43	75.68	88.17	92.86	78.38	87.10	98.21	89.19	94.62
HD + L5 + Outer Ankle	96.43	83.78	91.40	83.93	54.05	72.04	92.86	75.68	86.02
HD + L5 + Lower Body	91.07	78.38	86.02	92.86	78.38	87.10	92.86	75.68	86.02
C7 + L5 + Inner Ankle	89.29	83.78	87.10	91.07	83.78	88.17	94.64	97.30	95.70
C7 + L5 + Outer Ankle	91.07	86.49	89.25	80.36	56.76	70.97	92.86	81.08	88.17
C7 + L5 + Lower Body	87.50	81.08	84.95	83.93	56.76	73.12	96.43	97.30	96.77
Upper Body + Inner Ankle	94.64	91.89	93.55	94.64	78.38	88.17	100.00	94.59	97.85
Upper Body + Outer Ankle	98.21	94.59	96.77	91.07	81.08	87.10	92.86	81.08	88.17
Full Body	94.64	91.89	93.55	94.64	83.78	90.32	94.64	83.78	90.32

**Table A2:** Classification accuracy (%) based on the raters’ observation.

Raters	Accuracy	Sensitivity	Specificity
Rater 1	77.38	71.43	81.63
Rater 2	75.00	80.00	71.43
