# Supplementary material for: Turning Detection During Gait: Algorithm Validation and Influence of Sensor Location and Turning Characteristics in the Classification of Parkinson’s Disease
Source: Sensors (Basel). 2020 Sep 19;20(18):5377. doi: 10.3390/s20185377 (PMC7570702; doi:10.3390/s20185377)
Supplement: Supplementary file 1 [file sensors-20-05377-s001.pdf]

Turning Detection During Gait: Algorithm Validation and Influence of Sensor Location and Turning Characteristics in the Classification of Parkinson's Disease

Rana Zia Ur Rehman [1,2], Philipp Klocke [2,3], Sofia Hryniv [2,4], Brook Galna [1,2,5], Lynn Rochester [1,2,6], Silvia Del Din [1,2] and Lisa Alcock [1,2*]

[1] Translational and Clinical Research Institute, Newcastle University, Newcastle upon Tyne, UK; Rana.zia-ur-Rehman@newcastle.ac.uk (R.Z.U.R.); Brook.Galna@newcastle.ac.uk (B.G.); Lynn.Rochester@newcastle.ac.uk (L.R.); Silvia.Del-Din@newcastle.ac.uk (S.D.D.);

[2] Clinical Ageing Research Unit, Campus for Ageing and Vitality, Newcastle University, Newcastle upon Tyne, UK; philipp-klocke@gmx.de (P.K.); sh959@cam.ac.uk (S.H.)

[3] Faculty of Medicine, University of Southampton, University Road, Southampton, UK

[4] Department of Psychology, University of Cambridge, Cambridge, UK

[5] School of Biomedical, Nutritional and Sport Sciences, Newcastle University, Newcastle upon Tyne, UK

[6] The Newcastle upon Tyne NHS Foundation Trust, Newcastle upon Tyne, UK

* Correspondence: Lisa.Alcock@newcastle.ac.uk; Tel.: +44–191–208–1283

Supplementary Material

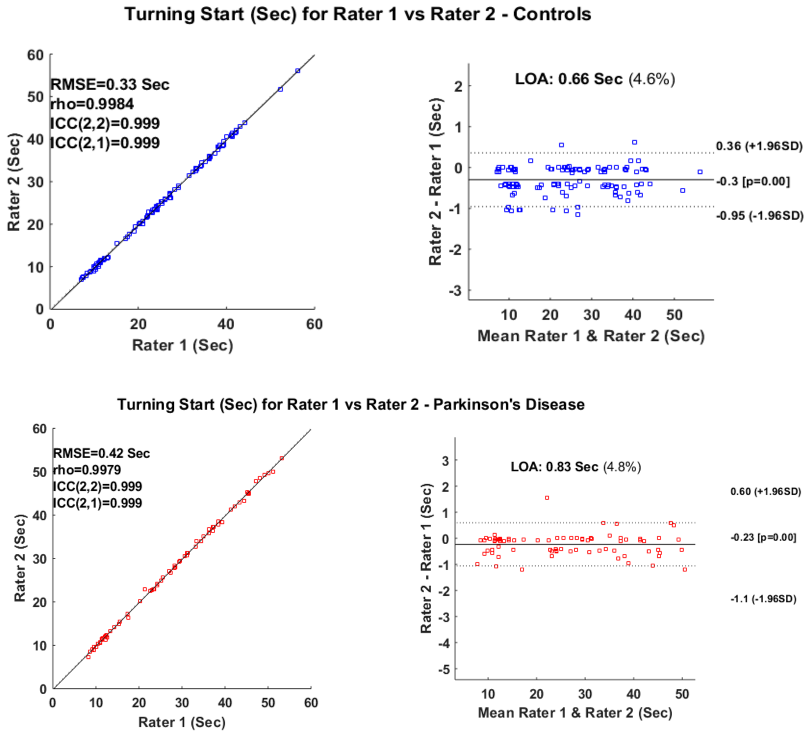

(a) Rater 1 vs. Rater 2—Turn Start.

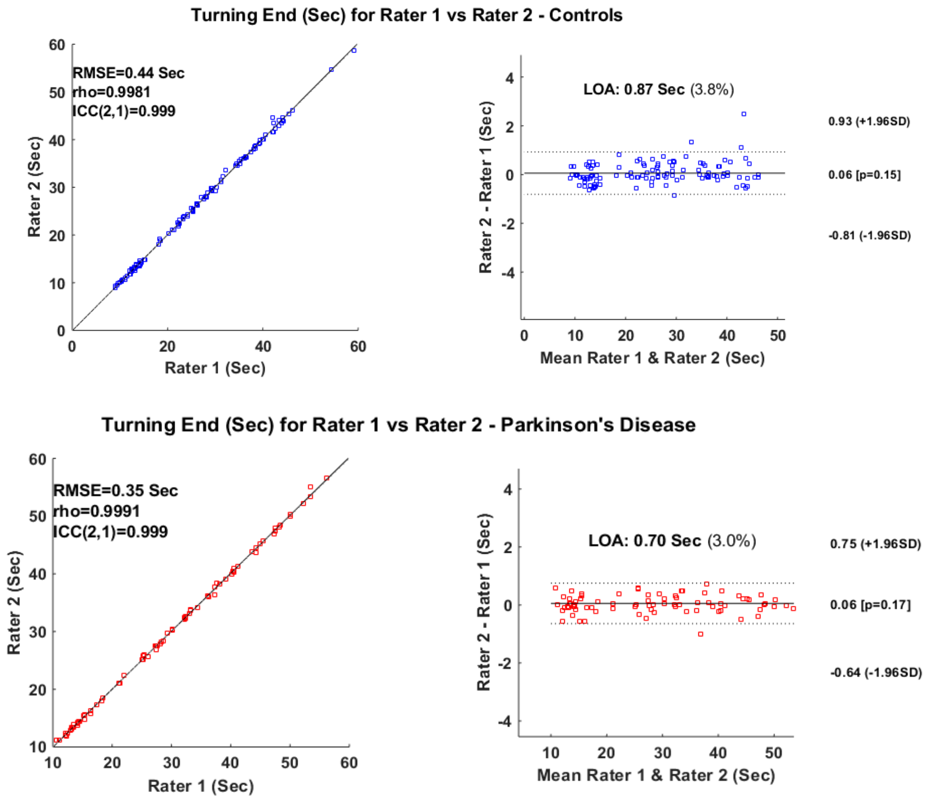

(b) Rater 1 vs. Rater 2—Turn End.

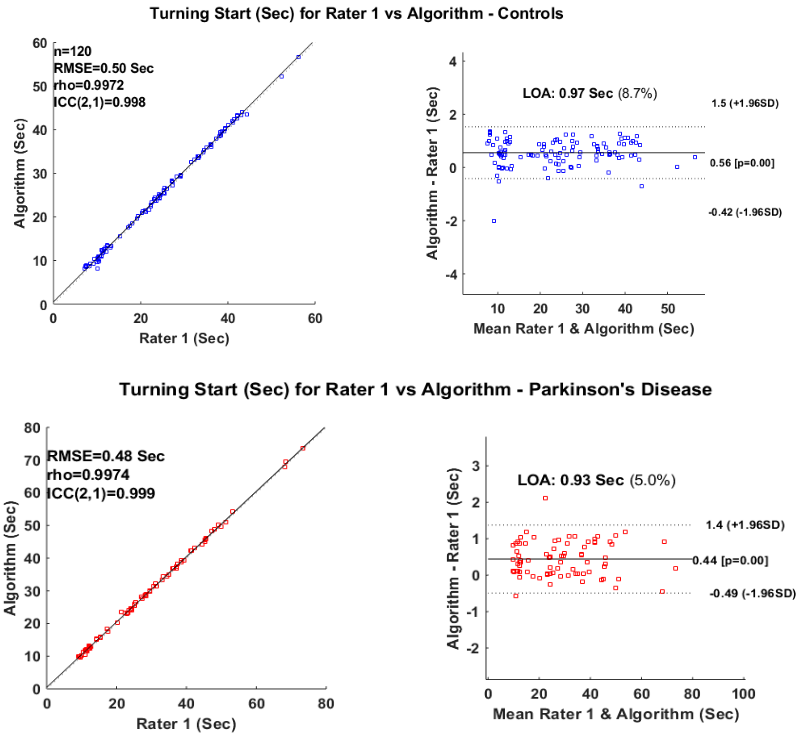

(c) Rater 1 vs. Algorithm—Turn Start.

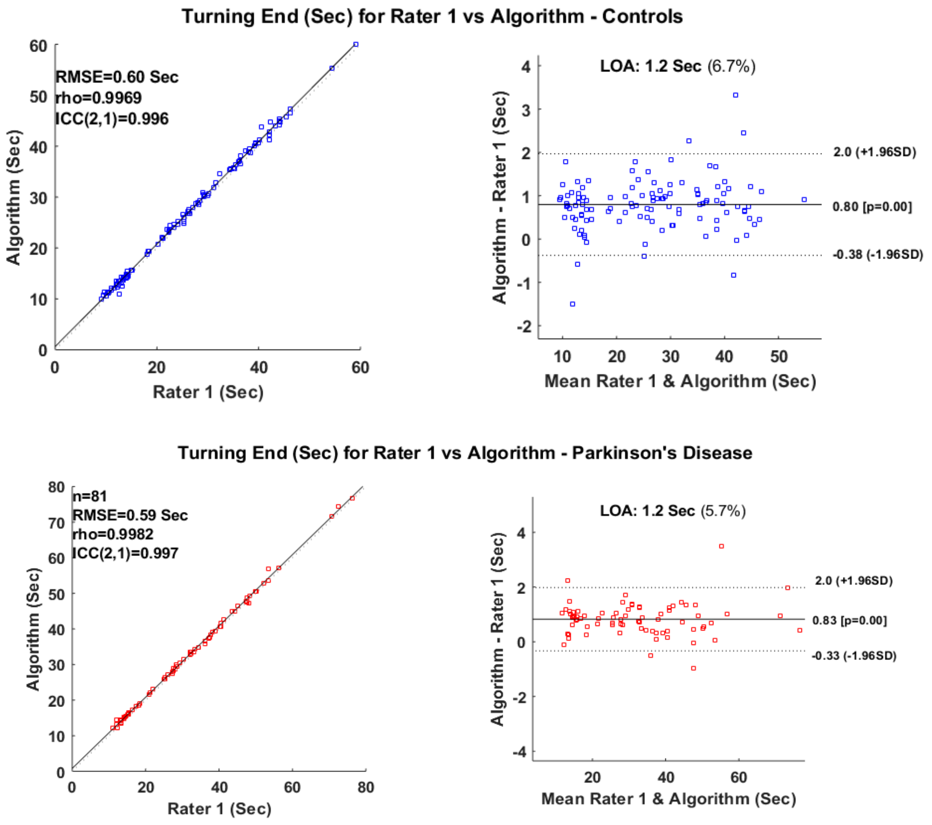

(d) Rater 1 vs. Algorithm—Turn End.

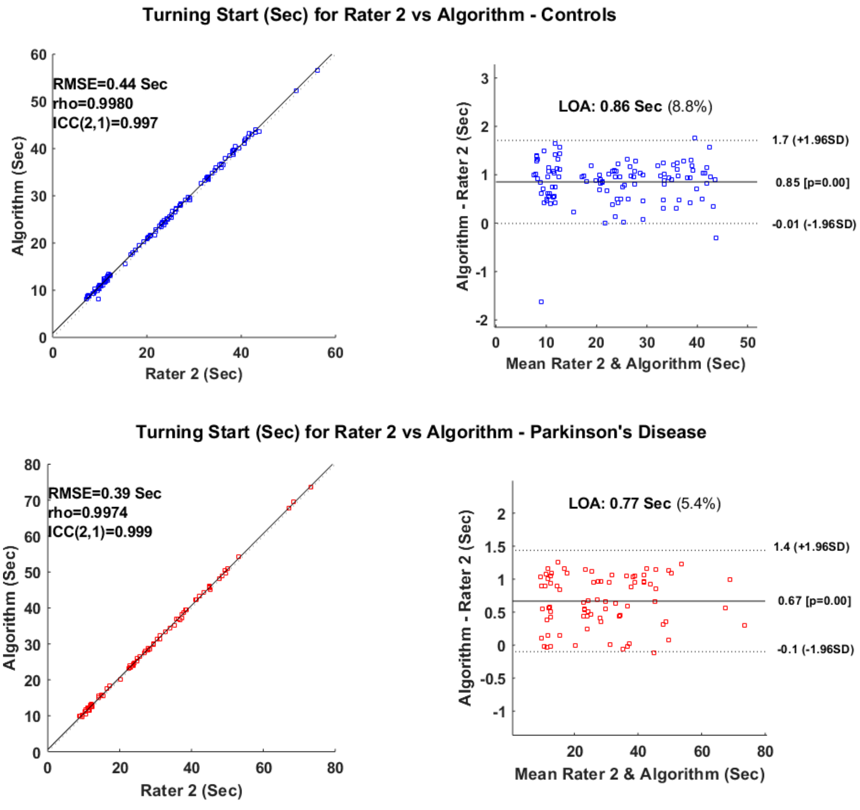

(e) Rater 2 vs. Algorithm—Turn Start.

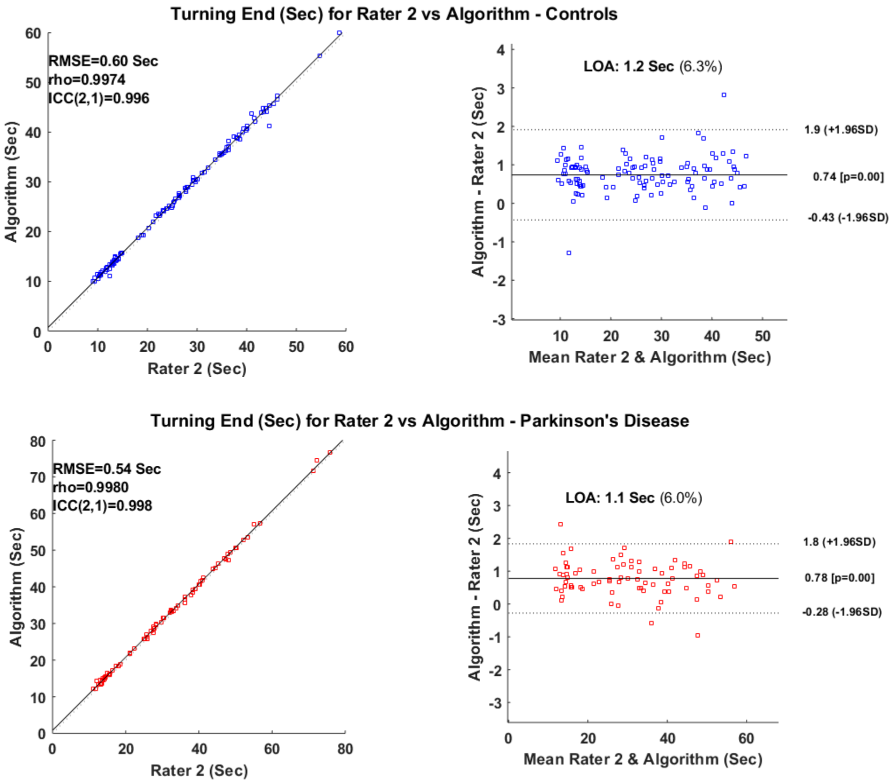

(f) Rater 2 vs. Algorithm—Turn End.

Figure S1. Scatter plots and Bland Altman plots for validation of the turning start and turning end.

Table S1. Statistical analysis of turning characteristics from the lower back (L5), neck (C7), head (HD), inner ankle (inner), outer ankle (outer).

Turning Characteristics	Control Group	PD Group	Student t-test	Mann Whitney
**Turning Characteristics from Lower Back (L5)**				
Number of Turns	3.48 ± 0.54	3.22 ± 0.82	0.062	**0.043**
Angle of Turn-Max	188.15 ± 45.69	176.93 ± 29.30	0.189	**0.004**
Angle of Turn-Min	154.61 ± 21.49	157.61 ± 32.06	0.591	0.553
Angle of Turn-Average	172.59 ± 22.99	168.37 ± 29.33	0.440	0.056
Angle of Turn-Variability	15.99 ± 22.75	9.56 ± 8.02	0.102	**0.034**
No. of Direction of Turns-Right	2.30 ± 1.11	2.49 ± 1.15	0.445	0.471
No. of Direction of Turns-Left	1.18 ± 1.03	0.73 ± 1.12	**0.050**	**0.020**
Duration of Turn-Max	3.04 ± 1.05	3.88 ± 1.41	**0.001**	**0.001**
Duration of Turn-Min	1.94 ± 0.54	2.70 ± 1.04	**< 0.0001**	**< 0.0001**
Duration of Turn-Average	2.46 ± 0.64	3.25 ± 1.03	**< 0.0001**	**< 0.0001**
Duration of Turn-Variability	0.53 ± 0.47	0.58 ± 0.63	0.629	0.854
Angular Velocity-Average	75.85 ± 17.53	56.74 ± 14.43	**< 0.0001**	**< 0.0001**
Angular Velocity-Variability	13.46 ± 8.24	8.98 ± 7.47	**0.009**	**0.003**
Peak Angular Velocity-Average	175.72 ± 38.44	136.68 ± 34.04	**< 0.0001**	**< 0.0001**
Peak Angular Velocity-Variability	24.47 ± 13.12	16.30 ± 11.79	**0.003**	**0.002**
Angular Velocity Start-Average	31.84 ± 16.08	24.63 ± 11.09	**0.020**	**0.030**
Angular Velocity Start-Variability	15.66 ± 9.51	10.11 ± 6.56	**0.003**	**0.002**

Angular Velocity End-Average	13.67 ± 7.48	10.39 ± 9.01	0.060	**0.001**
Angular Velocity End-Variability	7.93 ± 5.39	4.80 ± 6.20	**0.012**	**0.000**
Angular Velocity Middle-Average	107.62 ± 37.82	72.77 ± 30.48	**< 0.0001**	**< 0.0001**
Angular Velocity Middle-Variability	38.55 ± 24.35	25.85 ± 16.91	**0.007**	**0.016**
RMS-Acceleration-ML	0.14 ± 0.03	0.12 ± 0.03	**0.000**	**0.000**
RMS-Acceleration-Start-ML	0.16 ± 0.09	0.16 ± 0.08	0.989	0.751
RMS-Acceleration-Mid-ML	0.14 ± 0.04	0.12 ± 0.07	**0.044**	**0.004**
RMS-Acceleration-End-ML	0.11 ± 0.04	0.08 ± 0.05	**0.013**	**0.002**
RMS-Angular Velocity-ML	0.25 ± 0.07	0.21 ± 0.06	**0.003**	**0.001**
RMS-Angular Velocity-Start-ML	0.26 ± 0.14	0.22 ± 0.12	0.114	0.121
RMS-Angular Velocity-Mid-ML	0.23 ± 0.10	0.19 ± 0.13	0.095	**0.006**
RMS-Angular Velocity-End-ML	0.15 ± 0.06	0.12 ± 0.07	**0.009**	**0.000**
RMS of Jerk-ML	3.92 ± 1.55	3.25 ± 1.29	**0.031**	**0.029**
Max of Jerk-ML	16.14 ± 7.61	14.79 ± 5.96	0.364	0.538
Min of Jerk-ML	16.43 ± 7.75	15.00 ± 6.79	0.363	0.403
Range of Jerk-ML	32.58 ± 15.07	29.79 ± 12.19	0.351	0.522
RMS of Jerk-Start-ML	6.00 ± 4.46	5.54 ± 3.40	0.596	0.860
RMS of Jerk-Mid-ML	2.83 ± 1.45	3.06 ± 3.80	0.677	0.072
RMS of Jerk-End-ML	2.11 ± 1.86	1.77 ± 1.80	0.380	0.077
RMS of Angular Acceleration-ML	6.62 ± 3.17	4.89 ± 2.92	**0.009**	**0.003**
Max of Angular Acceleration-ML	29.22 ± 16.03	22.70 ± 18.94	0.078	**0.019**
Min of Angular Acceleration-ML	30.21 ± 16.62	24.40 ± 16.52	0.102	0.051
Range of Angular Acceleration-ML	59.44 ± 31.78	47.11 ± 34.98	0.082	**0.026**
RMS of Angular Acceleration-Start-ML	11.13 ± 8.39	8.08 ± 6.29	0.063	**0.037**
RMS of Angular Acceleration-Mid-ML	4.61 ± 3.15	3.89 ± 4.73	0.381	**0.021**
RMS of Angular Acceleration-End-ML	3.21 ± 1.95	2.71 ± 3.05	0.338	**0.024**
RMS-Acceleration-VT	0.11 ± 0.04	0.09 ± 0.04	**0.018**	**0.003**
RMS-Acceleration-Start-VT	0.18 ± 0.10	0.15 ± 0.08	0.223	0.421
RMS-Acceleration-Mid-VT	0.07 ± 0.04	0.07 ± 0.07	0.924	**0.030**
RMS-Acceleration-End-VT	0.07 ± 0.04	0.06 ± 0.08	0.542	**0.020**
RMS-Angular Velocity-VT	1.49 ± 0.36	1.07 ± 0.32	**< 0.0001**	**< 0.0001**
RMS-Angular Velocity-Start-VT	0.49 ± 0.26	0.36 ± 0.18	**0.009**	**0.018**
RMS-Angular Velocity-Mid-VT	1.81 ± 0.66	1.17 ± 0.55	**< 0.0001**	**< 0.0001**
RMS-Angular Velocity-End-VT	0.24 ± 0.13	0.18 ± 0.14	**0.039**	**0.002**
RMS of Jerk-VT	4.38 ± 2.31	3.22 ± 1.49	**0.008**	**0.004**
Max of Jerk-VT	21.90 ± 12.25	17.94 ± 9.81	0.103	0.054
Min of Jerk-VT	20.36 ± 12.81	16.31 ± 8.73	0.097	0.066
Range of Jerk-VT	42.25 ± 24.67	34.25 ± 18.06	0.094	0.056
RMS of Jerk-Start-VT	7.11 ± 6.25	5.65 ± 4.07	0.214	0.458
RMS of Jerk-Mid-VT	2.49 ± 1.74	2.51 ± 2.68	0.964	0.263
RMS of Jerk-End-VT	2.27 ± 2.26	2.22 ± 4.77	0.943	0.094
RMS of Angular Acceleration-VT	9.28 ± 3.17	6.89 ± 3.44	**0.001**	**< 0.0001**
Max of Angular Acceleration-VT	38.95 ± 18.93	31.69 ± 18.49	0.071	**0.030**
Min of Angular Acceleration-VT	36.95 ± 14.31	27.07 ± 16.63	**0.003**	**< 0.0001**

Range of Angular Acceleration-VT	75.89 ± 30.52	58.76 ± 34.36	**0.013**	**0.001**
RMS of Angular Acceleration-Start-VT	17.19 ± 9.19	13.44 ± 7.25	**0.040**	**0.033**
RMS of Angular Acceleration-Mid-VT	6.98 ± 3.93	7.01 ± 9.75	0.986	**0.018**
RMS of Angular Acceleration-End-VT	6.22 ± 3.52	4.72 ± 4.06	0.063	**0.001**
RMS-Acceleration-AP	0.09 ± 0.03	0.08 ± 0.03	0.062	**0.002**
RMS-Acceleration-Start-AP	0.15 ± 0.07	0.12 ± 0.06	**0.013**	**0.011**
RMS-Acceleration-Mid-AP	0.07 ± 0.05	0.06 ± 0.05	0.378	**0.048**
RMS-Acceleration-End-AP	0.06 ± 0.03	0.07 ± 0.07	0.276	0.817
RMS-Angular Velocity-AP	0.41 ± 0.23	0.27 ± 0.14	**0.002**	**0.000**
RMS-Angular Velocity-Start-AP	0.31 ± 0.16	0.26 ± 0.12	0.129	0.189
RMS-Angular Velocity-Mid-AP	0.50 ± 0.36	0.28 ± 0.17	**0.001**	**0.000**
RMS-Angular Velocity-End-AP	0.15 ± 0.08	0.10 ± 0.09	**0.007**	**0.000**
RMS of Jerk-AP	3.22 ± 1.44	2.34 ± 1.05	**0.002**	**0.000**
Max of Jerk-AP	15.65 ± 8.24	11.88 ± 7.98	**0.031**	**0.004**
Min of Jerk-AP	16.23 ± 7.87	12.99 ± 7.00	**0.046**	**0.009**
Range of Jerk-AP	31.88 ± 15.57	24.87 ± 14.54	**0.032**	**0.003**
RMS of Jerk-Start-AP	5.63 ± 4.27	3.96 ± 2.91	**0.040**	**0.023**
RMS of Jerk-Mid-AP	1.87 ± 1.44	1.72 ± 2.00	0.677	0.072
RMS of Jerk-End-AP	1.54 ± 1.26	1.81 ± 4.91	0.686	**0.029**
RMS of Angular Acceleration-AP	6.08 ± 2.36	4.45 ± 1.76	**0.001**	**0.000**
Max of Angular Acceleration-AP	25.35 ± 11.19	19.55 ± 8.41	**0.008**	**0.009**
Min of Angular Acceleration-AP	23.80 ± 10.03	19.26 ± 8.24	**0.024**	**0.017**
Range of Angular Acceleration-AP	49.15 ± 20.57	38.80 ± 15.91	**0.011**	**0.009**
RMS of Angular Acceleration-Start-AP	10.14 ± 5.80	8.00 ± 4.32	0.057	0.129
RMS of Angular Acceleration-Mid-AP	4.46 ± 2.53	3.50 ± 3.19	0.112	**0.005**
RMS of Angular Acceleration-End-AP	3.25 ± 2.52	2.37 ± 2.68	0.112	**0.001**
RMS-Acceleration-R	0.21 ± 0.05	0.17 ± 0.05	**0.003**	**0.001**
RMS-Acceleration-Start-R	0.30 ± 0.13	0.27 ± 0.10	0.234	0.317
RMS-Acceleration-Mid-R	0.19 ± 0.06	0.17 ± 0.10	0.177	**0.002**
RMS-Acceleration-End-R	0.15 ± 0.05	0.14 ± 0.11	0.394	**0.010**
RMS-Angular Velocity-R	1.58 ± 0.37	1.13 ± 0.33	**< 0.0001**	**< 0.0001**
RMS-Angular Velocity-Start-R	0.69 ± 0.29	0.54 ± 0.19	**0.008**	**0.020**
RMS-Angular Velocity-Mid-R	1.93 ± 0.68	1.25 ± 0.55	**< 0.0001**	**< 0.0001**
RMS-Angular Velocity-End-R	0.35 ± 0.14	0.26 ± 0.17	**0.006**	**0.000**
RMS of Jerk-R	4.47 ± 2.06	3.31 ± 1.35	**0.003**	**0.002**
Max of Jerk-R	22.84 ± 13.41	18.43 ± 10.18	0.093	0.078
Min of Jerk-R	23.31 ± 10.98	19.45 ± 9.60	0.084	**0.045**
Range of Jerk-R	46.15 ± 23.92	37.88 ± 19.35	0.082	**0.047**
RMS of Jerk-Start-R	6.83 ± 4.93	5.89 ± 3.97	0.332	0.394
RMS of Jerk-Mid-R	2.63 ± 1.50	2.79 ± 3.29	0.739	0.173
RMS of Jerk-End-R	2.18 ± 1.91	2.42 ± 5.53	0.767	**0.039**
RMS of Angular Acceleration-R	8.73 ± 2.74	6.44 ± 2.71	**0.000**	**< 0.0001**
Max of Angular Acceleration-R	38.93 ± 16.37	29.34 ± 15.59	**0.006**	**0.001**

Min of Angular Acceleration-R	34.98 ± 12.95	28.00 ± 15.22	**0.020**	**0.002**
Range of Angular Acceleration-R	73.91 ± 26.96	57.34 ± 29.71	**0.007**	**0.001**
RMS of Angular Acceleration-Start-R	14.30 ± 7.35	10.86 ± 5.50	**0.017**	**0.020**
RMS of Angular Acceleration-Mid-R	6.68 ± 3.72	6.12 ± 7.02	0.621	**0.010**
RMS of Angular Acceleration-End-R	4.78 ± 2.60	3.72 ± 3.23	0.084	**0.001**
**Turning Characteristics from Neck (C7)**				
Number of Turns	3.84 ± 1.06	4.24 ± 2.17	0.234	0.539
Angle of Turn-Max	118.55 ± 23.34	106.88 ± 27.47	**0.030**	**0.047**
Angle of Turn-Min	88.74 ± 35.01	77.50 ± 39.77	0.155	0.216
Angle of Turn-Average	105.35 ± 25.05	92.65 ± 31.50	**0.034**	0.065
Angle of Turn-Variability	13.51 ± 11.68	12.87 ± 12.76	0.803	0.231
No. of Direction of Turns-Right	2.52 ± 1.18	3.11 ± 1.68	**0.049**	0.092
No. of Direction of Turns-Left	1.32 ± 1.18	1.14 ± 1.62	0.522	0.170
Duration of Turn-Max	3.03 ± 0.75	3.82 ± 1.56	**0.002**	**0.009**
Duration of Turn-Min	1.80 ± 0.59	2.17 ± 0.82	**0.012**	**0.012**
Duration of Turn-Average	2.37 ± 0.54	2.86 ± 0.80	**0.001**	**0.000**
Duration of Turn-Variability	0.57 ± 0.33	0.73 ± 0.55	0.073	0.348
Angular Velocity-Average	47.31 ± 12.71	34.06 ± 10.57	**< 0.0001**	**< 0.0001**
Angular Velocity-Variability	9.25 ± 6.01	6.86 ± 4.57	**0.042**	**0.047**
Peak Angular Velocity-Average	109.48 ± 26.93	82.82 ± 25.83	**< 0.0001**	**< 0.0001**
Peak Angular Velocity-Variability	16.18 ± 9.26	15.11 ± 10.39	0.603	0.538
Angular Velocity Start-Average	11.95 ± 6.50	10.48 ± 4.92	0.242	0.352
Angular Velocity Start-Variability	6.07 ± 4.18	4.97 ± 3.76	0.199	0.143
Angular Velocity End-Average	7.13 ± 3.73	6.58 ± 3.91	0.498	0.241
Angular Velocity End-Variability	3.90 ± 4.53	3.55 ± 2.57	0.676	0.984
Angular Velocity Middle-Average	70.25 ± 25.66	49.45 ± 23.15	**0.000**	**0.000**
Angular Velocity Middle-Variability	27.48 ± 12.84	19.56 ± 11.41	**0.003**	**0.003**
RMS-Acceleration-ML	0.15 ± 0.03	0.13 ± 0.04	**0.011**	**0.001**
RMS-Acceleration-Start-ML	0.16 ± 0.07	0.15 ± 0.06	0.608	0.811
RMS-Acceleration-Mid-ML	0.14 ± 0.06	0.13 ± 0.05	0.285	0.507
RMS-Acceleration-End-ML	0.12 ± 0.05	0.11 ± 0.06	0.449	0.280
RMS-Angular Velocity-ML	0.25 ± 0.08	0.20 ± 0.09	**0.009**	**0.000**
RMS-Angular Velocity-Start-ML	0.18 ± 0.07	0.16 ± 0.09	0.209	0.070
RMS-Angular Velocity-Mid-ML	0.23 ± 0.11	0.17 ± 0.08	**0.009**	**0.004**
RMS-Angular Velocity-End-ML	0.15 ± 0.06	0.12 ± 0.07	**0.041**	**0.007**
RMS of Jerk-ML	2.16 ± 0.85	1.81 ± 0.75	**0.041**	**0.020**
Max of Jerk-ML	8.98 ± 3.94	7.80 ± 3.50	0.142	0.079
Min of Jerk-ML	8.34 ± 4.14	7.18 ± 3.36	0.160	0.139
Range of Jerk-ML	17.32 ± 7.67	14.98 ± 6.39	0.128	0.094
RMS of Jerk-Start-ML	2.34 ± 2.22	2.09 ± 0.83	0.512	0.497
RMS of Jerk-Mid-ML	1.85 ± 1.36	1.42 ± 0.95	0.097	**0.039**
RMS of Jerk-End-ML	1.08 ± 0.76	0.99 ± 0.54	0.532	0.698
RMS of Angular Acceleration-ML	5.27 ± 2.76	4.16 ± 2.59	0.056	**0.020**

Max of Angular Acceleration-ML	20.48 ± 13.78	16.73 ± 12.06	0.181	0.154
Min of Angular Acceleration-ML	23.06 ± 13.49	19.08 ± 12.53	0.156	0.103
Range of Angular Acceleration-ML	43.54 ± 26.72	35.81 ± 23.83	0.158	0.154
RMS of Angular Acceleration-Start-ML	5.70 ± 3.96	4.75 ± 2.91	0.212	0.277
RMS of Angular Acceleration-Mid-ML	4.35 ± 3.58	2.98 ± 2.93	0.055	**0.011**
RMS of Angular Acceleration-End-ML	2.98 ± 2.95	2.41 ± 1.78	0.289	0.231
RMS-Acceleration-VT	0.12 ± 0.03	0.11 ± 0.04	0.280	**0.040**
RMS-Acceleration-Start-VT	0.13 ± 0.06	0.13 ± 0.06	0.765	0.757
RMS-Acceleration-Mid-VT	0.09 ± 0.04	0.09 ± 0.04	0.973	0.793
RMS-Acceleration-End-VT	0.09 ± 0.06	0.07 ± 0.05	0.103	**0.024**
RMS-Angular Velocity-VT	0.94 ± 0.25	0.65 ± 0.20	**< 0.0001**	**< 0.0001**
RMS-Angular Velocity-Start-VT	0.18 ± 0.10	0.16 ± 0.07	0.184	0.274
RMS-Angular Velocity-Mid-VT	1.17 ± 0.44	0.79 ± 0.38	**< 0.0001**	**< 0.0001**
RMS-Angular Velocity-End-VT	0.12 ± 0.06	0.12 ± 0.06	0.652	0.463
RMS of Jerk-VT	3.73 ± 1.80	2.84 ± 1.15	**0.009**	**0.004**
Max of Jerk-VT	17.81 ± 9.61	15.01 ± 6.97	0.130	0.168
Min of Jerk-VT	15.37 ± 8.64	12.46 ± 5.54	0.073	0.069
Range of Jerk-VT	33.18 ± 17.94	27.47 ± 12.17	0.094	0.097
RMS of Jerk-Start-VT	3.99 ± 3.18	3.55 ± 1.81	0.453	0.953
RMS of Jerk-Mid-VT	2.64 ± 1.76	1.79 ± 1.19	**0.012**	**0.005**
RMS of Jerk-End-VT	1.89 ± 2.61	1.22 ± 0.62	0.131	0.291
RMS of Angular Acceleration-VT	4.35 ± 1.70	3.57 ± 1.77	**0.035**	**0.008**
Max of Angular Acceleration-VT	14.69 ± 7.44	13.71 ± 7.18	0.527	0.548
Min of Angular Acceleration-VT	13.15 ± 6.10	11.31 ± 6.07	0.157	0.061
Range of Angular Acceleration-VT	27.84 ± 12.83	25.01 ± 12.90	0.302	0.186
RMS of Angular Acceleration-Start-VT	5.94 ± 3.31	5.26 ± 2.60	0.301	0.522
RMS of Angular Acceleration-Mid-VT	3.94 ± 2.14	3.15 ± 1.82	0.070	**0.027**
RMS of Angular Acceleration-End-VT	3.26 ± 1.74	3.01 ± 1.78	0.513	0.194
RMS-Acceleration-AP	0.09 ± 0.03	0.08 ± 0.03	0.176	0.078
RMS-Acceleration-Start-AP	0.12 ± 0.06	0.12 ± 0.05	0.824	0.997
RMS-Acceleration-Mid-AP	0.07 ± 0.03	0.07 ± 0.03	0.295	0.183
RMS-Acceleration-End-AP	0.06 ± 0.04	0.06 ± 0.04	0.557	0.377
RMS-Angular Velocity-AP	1.11 ± 0.32	0.86 ± 0.36	**0.001**	**< 0.0001**
RMS-Angular Velocity-Start-AP	0.35 ± 0.19	0.32 ± 0.14	0.369	0.817
RMS-Angular Velocity-Mid-AP	1.40 ± 0.48	1.08 ± 0.45	**0.002**	**0.002**
RMS-Angular Velocity-End-AP	0.30 ± 0.19	0.27 ± 0.34	0.574	0.058
RMS of Jerk-AP	2.15 ± 0.93	1.62 ± 0.65	**0.004**	**0.001**
Max of Jerk-AP	8.08 ± 3.79	6.50 ± 2.86	**0.034**	**0.040**
Min of Jerk-AP	9.53 ± 5.50	6.99 ± 3.27	**0.013**	**0.006**
Range of Jerk-AP	17.62 ± 8.98	13.50 ± 5.95	**0.016**	**0.009**
RMS of Jerk-Start-AP	2.49 ± 1.70	1.95 ± 0.83	0.077	0.159
RMS of Jerk-Mid-AP	1.60 ± 0.87	1.18 ± 0.75	**0.016**	**0.005**
RMS of Jerk-End-AP	1.33 ± 1.53	0.91 ± 0.43	0.102	0.105

RMS of Angular Acceleration-AP	4.49 ± 1.66	3.55 ± 1.44	**0.006**	**0.003**
Max of Angular Acceleration-AP	13.78 ± 6.06	11.50 ± 4.94	0.060	0.060
Min of Angular Acceleration-AP	14.50 ± 7.10	12.21 ± 5.16	0.094	0.139
Range of Angular Acceleration-AP	28.28 ± 12.69	23.71 ± 9.86	0.067	0.075
RMS of Angular Acceleration-Start-AP	5.36 ± 3.64	4.49 ± 2.31	0.202	0.133
RMS of Angular Acceleration-Mid-AP	3.97 ± 1.87	3.00 ± 1.65	**0.012**	**0.001**
RMS of Angular Acceleration-End-AP	2.93 ± 1.42	2.55 ± 1.74	0.262	0.054
RMS-Acceleration-R	0.22 ± 0.04	0.20 ± 0.06	0.064	**0.003**
RMS-Acceleration-Start-R	0.25 ± 0.09	0.25 ± 0.08	0.937	0.928
RMS-Acceleration-Mid-R	0.20 ± 0.06	0.19 ± 0.06	0.316	0.284
RMS-Acceleration-End-R	0.17 ± 0.07	0.15 ± 0.08	0.173	**0.026**
RMS-Angular Velocity-R	1.49 ± 0.34	1.12 ± 0.35	**< 0.0001**	**< 0.0001**
RMS-Angular Velocity-Start-R	0.47 ± 0.19	0.42 ± 0.15	0.191	0.463
RMS-Angular Velocity-Mid-R	1.89 ± 0.57	1.40 ± 0.51	**< 0.0001**	**< 0.0001**
RMS-Angular Velocity-End-R	0.38 ± 0.19	0.35 ± 0.34	0.548	**0.036**
RMS of Jerk-R	2.84 ± 1.39	2.18 ± 0.84	**0.012**	**0.005**
Max of Jerk-R	13.42 ± 7.81	10.78 ± 4.52	0.066	0.097
Min of Jerk-R	13.22 ± 7.65	10.97 ± 5.16	0.121	0.168
Range of Jerk-R	26.64 ± 15.20	21.75 ± 9.50	0.085	0.139
RMS of Jerk-Start-R	2.96 ± 2.14	2.55 ± 1.21	0.299	0.781
RMS of Jerk-Mid-R	2.09 ± 1.27	1.43 ± 0.86	**0.007**	**0.003**
RMS of Jerk-End-R	1.53 ± 2.42	1.00 ± 0.53	0.194	0.112
RMS of Angular Acceleration-R	5.29 ± 1.90	4.28 ± 1.92	**0.014**	**0.004**
Max of Angular Acceleration-R	17.97 ± 8.90	16.03 ± 9.11	0.311	0.234
Min of Angular Acceleration-R	16.44 ± 7.07	15.14 ± 8.52	0.427	0.139
Range of Angular Acceleration-R	34.41 ± 15.34	31.17 ± 17.29	0.346	0.194
RMS of Angular Acceleration-Start-R	5.85 ± 3.53	5.01 ± 2.32	0.200	0.267
RMS of Angular Acceleration-Mid-R	4.76 ± 2.12	3.65 ± 1.84	**0.011**	**0.003**
RMS of Angular Acceleration-End-R	3.27 ± 1.63	2.98 ± 1.85	0.423	0.105
**Turning Characteristics from Head (HD)**				
Number of Turns	5.07 ± 1.70	5.78 ± 6.00	0.403	0.304
Angle of Turn-Max	178.71 ± 25.28	176.57 ± 13.78	0.639	0.463
Angle of Turn-Min	77.77 ± 54.36	92.00 ± 60.39	0.241	0.317
Angle of Turn-Average	134.51 ± 29.76	138.32 ± 32.34	0.561	0.477
Angle of Turn-Variability	45.52 ± 25.76	37.21 ± 27.57	0.142	0.082
No. of Direction of Turns-Right	3.38 ± 1.48	4.08 ± 2.81	0.118	0.344
No. of Direction of Turns-Left	1.70 ± 1.26	1.70 ± 3.49	0.990	0.084
Duration of Turn-Max	3.42 ± 0.94	4.15 ± 1.13	**0.001**	**0.001**
Duration of Turn-Min	1.50 ± 0.76	2.07 ± 1.02	**0.002**	**0.004**
Duration of Turn-Average	2.37 ± 0.60	3.02 ± 0.83	**< 0.0001**	**< 0.0001**
Duration of Turn-Variability	0.79 ± 0.41	0.87 ± 0.56	0.388	0.675
Angular Velocity-Average	58.27 ± 9.96	47.67 ± 10.40	**< 0.0001**	**< 0.0001**
Angular Velocity-Variability	16.67 ± 9.57	10.95 ± 5.98	**0.002**	**0.002**
Peak Angular Velocity-Average	165.01 ± 27.47	133.63 ± 28.37	**< 0.0001**	**< 0.0001**

Peak Angular Velocity-Variability	41.56 ± 19.02	35.38 ± 20.23	0.138	0.125
Angular Velocity Start-Average	11.15 ± 5.30	9.73 ± 6.13	0.238	0.079
Angular Velocity Start-Variability	8.20 ± 6.21	5.63 ± 4.34	**0.031**	**0.033**
Angular Velocity End-Average	8.09 ± 3.91	7.28 ± 5.28	0.398	0.127
Angular Velocity End-Variability	5.28 ± 3.80	4.33 ± 3.88	0.242	0.072
Angular Velocity Middle-Average	92.68 ± 29.75	81.63 ± 27.67	0.075	0.088
Angular Velocity Middle-Variability	50.59 ± 21.39	41.43 ± 21.04	**0.045**	**0.039**
RMS-Acceleration-ML	0.15 ± 0.03	0.13 ± 0.05	0.078	**0.004**
RMS-Acceleration-Start-ML	0.14 ± 0.05	0.12 ± 0.07	0.181	**0.047**
RMS-Acceleration-Mid-ML	0.14 ± 0.05	0.13 ± 0.06	0.638	0.328
RMS-Acceleration-End-ML	0.10 ± 0.03	0.09 ± 0.06	0.444	**0.039**
RMS-Angular Velocity-ML	0.37 ± 0.12	0.27 ± 0.15	**0.000**	**< 0.0001**
RMS-Angular Velocity-Start-ML	0.24 ± 0.12	0.20 ± 0.17	0.197	**0.009**
RMS-Angular Velocity-Mid-ML	0.35 ± 0.18	0.26 ± 0.18	**0.027**	**0.010**
RMS-Angular Velocity-End-ML	0.29 ± 0.17	0.18 ± 0.15	**0.004**	**< 0.0001**
RMS of Jerk-ML	1.93 ± 0.54	1.80 ± 0.83	0.377	**0.023**
Max of Jerk-ML	7.15 ± 2.78	6.68 ± 3.50	0.473	0.148
Min of Jerk-ML	6.20 ± 2.16	6.18 ± 3.27	0.969	0.538
Range of Jerk-ML	13.35 ± 4.63	12.86 ± 6.60	0.673	0.219
RMS of Jerk-Start-ML	1.72 ± 0.86	1.72 ± 1.06	0.997	0.468
RMS of Jerk-Mid-ML	1.73 ± 0.72	1.77 ± 0.91	0.792	0.885
RMS of Jerk-End-ML	1.14 ± 0.76	1.07 ± 0.70	0.650	0.250
RMS of Angular Acceleration-ML	4.91 ± 2.14	4.23 ± 1.73	0.109	0.078
Max of Angular Acceleration-ML	15.12 ± 7.66	14.13 ± 5.69	0.502	0.635
Min of Angular Acceleration-ML	16.40 ± 9.40	16.44 ± 6.73	0.982	0.473
Range of Angular Acceleration-ML	31.52 ± 16.84	30.57 ± 12.00	0.767	0.762
RMS of Angular Acceleration-Start-ML	4.86 ± 2.47	4.34 ± 2.39	0.316	0.365
RMS of Angular Acceleration-Mid-ML	4.19 ± 2.73	2.99 ± 1.45	**0.016**	**0.008**
RMS of Angular Acceleration-End-ML	3.70 ± 2.77	2.90 ± 1.69	0.118	0.058
RMS-Acceleration-VT	0.12 ± 0.04	0.10 ± 0.03	**0.010**	**0.014**
RMS-Acceleration-Start-VT	0.14 ± 0.07	0.12 ± 0.04	0.065	0.194
RMS-Acceleration-Mid-VT	0.09 ± 0.05	0.08 ± 0.04	0.104	0.148
RMS-Acceleration-End-VT	0.09 ± 0.05	0.07 ± 0.03	**0.021**	0.053
RMS-Angular Velocity-VT	1.27 ± 0.21	0.99 ± 0.23	**< 0.0001**	**< 0.0001**
RMS-Angular Velocity-Start-VT	0.21 ± 0.14	0.17 ± 0.10	0.086	**0.016**
RMS-Angular Velocity-Mid-VT	1.56 ± 0.47	1.33 ± 0.46	**0.024**	**0.036**
RMS-Angular Velocity-End-VT	0.19 ± 0.16	0.16 ± 0.10	0.336	0.152
RMS of Jerk-VT	3.81 ± 1.51	3.27 ± 1.05	0.064	0.121
Max of Jerk-VT	16.30 ± 7.47	16.38 ± 6.64	0.960	0.721
Min of Jerk-VT	16.10 ± 7.26	14.57 ± 4.81	0.261	0.580
Range of Jerk-VT	32.40 ± 14.52	30.95 ± 11.07	0.605	0.978
RMS of Jerk-Start-VT	3.84 ± 2.40	3.35 ± 2.00	0.309	0.247

RMS of Jerk-Mid-VT	2.79 ± 1.72	2.14 ± 1.14	**0.045**	0.054
RMS of Jerk-End-VT	2.40 ± 1.71	1.80 ± 0.87	0.051	**0.030**
RMS of Angular Acceleration-VT	5.61 ± 1.47	4.34 ± 1.91	**0.000**	**< 0.0001**
Max of Angular Acceleration-VT	16.08 ± 4.59	13.61 ± 4.63	**0.013**	**0.008**
Min of Angular Acceleration-VT	14.23 ± 3.83	11.57 ± 3.48	**0.001**	**0.001**
Range of Angular Acceleration-VT	30.30 ± 7.89	25.17 ± 7.78	**0.003**	**0.002**
RMS of Angular Acceleration-Start-VT	5.24 ± 2.48	4.56 ± 2.76	0.218	0.100
RMS of Angular Acceleration-Mid-VT	5.23 ± 2.25	4.26 ± 1.79	**0.030**	**0.012**
RMS of Angular Acceleration-End-VT	3.50 ± 1.71	3.19 ± 2.31	0.458	0.108
RMS-Acceleration-AP	0.19 ± 0.06	0.16 ± 0.07	**0.023**	**0.003**
RMS-Acceleration-Start-AP	0.16 ± 0.07	0.15 ± 0.08	0.486	0.260
RMS-Acceleration-Mid-AP	0.15 ± 0.07	0.14 ± 0.07	0.351	0.328
RMS-Acceleration-End-AP	0.18 ± 0.09	0.14 ± 0.08	**0.022**	**0.009**
RMS-Angular Velocity-AP	0.36 ± 0.18	0.29 ± 0.14	**0.046**	**0.010**
RMS-Angular Velocity-Start-AP	0.19 ± 0.12	0.17 ± 0.15	0.369	0.089
RMS-Angular Velocity-Mid-AP	0.41 ± 0.24	0.32 ± 0.17	0.065	0.052
RMS-Angular Velocity-End-AP	0.14 ± 0.07	0.12 ± 0.11	0.296	**0.006**
RMS of Jerk-AP	1.78 ± 0.65	1.57 ± 0.45	0.090	0.121
Max of Jerk-AP	6.03 ± 3.16	5.76 ± 1.94	0.645	0.891
Min of Jerk-AP	6.98 ± 3.22	6.61 ± 2.46	0.554	0.602
Range of Jerk-AP	13.01 ± 6.22	12.37 ± 4.12	0.583	0.841
RMS of Jerk-Start-AP	1.61 ± 0.75	1.62 ± 0.75	0.944	0.959
RMS of Jerk-Mid-AP	1.42 ± 0.70	1.19 ± 0.47	0.093	0.241
RMS of Jerk-End-AP	1.24 ± 1.03	0.95 ± 0.41	0.117	0.116
RMS of Angular Acceleration-AP	3.56 ± 1.22	2.97 ± 1.41	**0.034**	**0.001**
Max of Angular Acceleration-AP	11.39 ± 5.05	10.07 ± 4.74	0.209	0.123
Min of Angular Acceleration-AP	11.07 ± 4.89	9.70 ± 4.27	0.170	0.069
Range of Angular Acceleration-AP	22.46 ± 9.65	19.78 ± 8.60	0.174	0.110
RMS of Angular Acceleration-Start-AP	3.33 ± 1.43	2.99 ± 2.06	0.347	0.061
RMS of Angular Acceleration-Mid-AP	3.36 ± 1.57	2.46 ± 1.29	**0.005**	**0.000**
RMS of Angular Acceleration-End-AP	2.17 ± 1.14	1.83 ± 1.20	0.172	**0.023**
RMS-Acceleration-R	0.28 ± 0.06	0.24 ± 0.08	**0.007**	**0.000**
RMS-Acceleration-Start-R	0.29 ± 0.08	0.26 ± 0.09	0.052	**0.008**
RMS-Acceleration-Mid-R	0.25 ± 0.07	0.23 ± 0.08	0.202	0.069
RMS-Acceleration-End-R	0.25 ± 0.09	0.20 ± 0.10	**0.033**	**0.006**
RMS-Angular Velocity-R	1.39 ± 0.23	1.08 ± 0.27	**< 0.0001**	**< 0.0001**
RMS-Angular Velocity-Start-R	0.42 ± 0.19	0.34 ± 0.24	0.067	**0.001**
RMS-Angular Velocity-Mid-R	1.71 ± 0.49	1.44 ± 0.47	**0.010**	**0.010**
RMS-Angular Velocity-End-R	0.42 ± 0.21	0.30 ± 0.22	**0.012**	**< 0.0001**
RMS of Jerk-R	2.80 ± 1.05	2.40 ± 0.81	0.056	0.058
Max of Jerk-R	13.45 ± 5.80	12.14 ± 4.12	0.238	0.527
Min of Jerk-R	12.66 ± 6.08	11.89 ± 4.38	0.504	0.885
Range of Jerk-R	26.11 ± 11.66	24.03 ± 8.23	0.348	0.698

RMS of Jerk-Start-R	2.71 ± 1.60	2.30 ± 1.19	0.190	0.298
RMS of Jerk-Mid-R	2.13 ± 1.20	1.77 ± 0.74	0.113	0.250
RMS of Jerk-End-R	1.68 ± 1.39	1.43 ± 0.78	0.319	0.340
RMS of Angular Acceleration-R	6.18 ± 1.77	4.72 ± 2.08	**0.001**	**< 0.0001**
Max of Angular Acceleration-R	19.68 ± 6.61	15.74 ± 5.71	**0.004**	**0.002**
Min of Angular Acceleration-R	15.85 ± 6.12	14.12 ± 5.24	0.162	0.068
Range of Angular Acceleration-R	35.53 ± 12.19	29.85 ± 10.66	**0.023**	**0.009**
RMS of Angular Acceleration-Start-R	5.44 ± 2.45	4.36 ± 2.72	0.051	**0.008**
RMS of Angular Acceleration-Mid-R	5.51 ± 2.56	4.30 ± 2.03	**0.018**	**0.010**
RMS of Angular Acceleration-End-R	3.79 ± 2.21	2.95 ± 1.84	0.059	**0.037**
**Turning Characteristics from Inner Turning Ankle**				
Number of Transitions/Steps	2.85 ± 1.61	3.51 ± 2.31	0.106	0.079
Angle of Turn-Average	145.97 ± 35.78	127.97 ± 44.38	**0.034**	**0.040**
Angle of Turn-Variability	47.92 ± 24.64	44.47 ± 28.63	0.537	0.527
Duration of Turn-Average	0.69 ± 0.19	0.63 ± 0.19	0.125	0.152
Duration of Turn-Variability	0.24 ± 0.12	0.22 ± 0.13	0.551	0.602
Angular Velocity-Average	382.37 ± 407.35	365.59 ± 264.36	0.825	0.613
Angular Velocity-Variability	264.91 ± 431.29	306.24 ± 422.94	0.650	0.234
Peak Angular Velocity-Average	16,671.21 ± 9,437.05	11,924.05 ± 9,287.73	**0.019**	**0.014**
Peak Angular Velocity-Variability	12,290.51 ± 7,040.74	10,213.44 ± 7,179.51	0.170	0.313
RMS-Acceleration-ML	0.39 ± 0.12	0.32 ± 0.10	**0.009**	**0.013**
RMS-Angular Velocity-ML	0.81 ± 0.34	0.72 ± 0.44	0.266	**0.036**
RMS of Jerk-ML	15.30 ± 5.87	13.36 ± 5.19	0.105	0.079
Max of Jerk-ML	46.79 ± 19.58	38.40 ± 17.44	**0.038**	**0.020**
Min of Jerk-ML	41.56 ± 17.08	35.43 ± 12.40	0.063	0.056
Range of Jerk-ML	88.35 ± 32.77	73.84 ± 23.39	**0.022**	**0.020**
RMS of Angular Acceleration-ML	11.20 ± 4.21	9.75 ± 4.56	0.120	0.073
Max of Angular Acceleration-ML	22.35 ± 7.68	18.57 ± 7.79	**0.023**	**0.012**
Min of Angular Acceleration-ML	28.34 ± 10.90	23.89 ± 9.87	**0.048**	**0.028**
Range of Angular Acceleration-ML	50.70 ± 16.53	42.46 ± 15.95	**0.019**	**0.004**
RMS-Acceleration-VT	0.29 ± 0.10	0.29 ± 0.11	0.764	0.799
RMS-Angular Velocity-VT	2.24 ± 0.57	1.79 ± 0.48	**0.000**	**< 0.0001**
RMS of Jerk-VT	8.20 ± 3.11	8.11 ± 3.93	0.904	0.473
Max of Jerk-VT	20.28 ± 8.44	19.22 ± 7.92	0.543	0.569
Min of Jerk-VT	23.91 ± 13.59	21.71 ± 9.14	0.390	0.348
Range of Jerk-VT	44.19 ± 20.47	40.92 ± 15.85	0.413	0.263
RMS of Angular Acceleration-VT	33.48 ± 8.93	29.62 ± 13.19	0.096	**0.001**
Max of Angular Acceleration-VT	74.49 ± 18.12	64.16 ± 24.85	**0.023**	**0.004**
Min of Angular Acceleration-VT	92.33 ± 26.14	75.17 ± 32.69	**0.006**	**0.000**
Range of Angular Acceleration-VT	166.81 ± 36.87	139.33 ± 49.67	**0.003**	**0.000**
RMS-Acceleration-AP	0.32 ± 0.10	0.27 ± 0.10	**0.023**	**0.006**

RMS-Angular Velocity-AP	1.62 ± 0.50	1.60 ± 0.61	0.881	0.984
RMS of Jerk-AP	10.20 ± 3.78	8.81 ± 4.56	0.114	**0.010**
Max of Jerk-AP	26.46 ± 9.74	22.43 ± 10.78	0.064	**0.020**
Min of Jerk-AP	30.54 ± 11.58	24.45 ± 11.67	**0.015**	**0.004**
Range of Jerk-AP	57.01 ± 18.93	46.88 ± 21.11	**0.018**	**0.004**
RMS of Angular Acceleration-AP	18.64 ± 6.22	17.24 ± 6.48	0.298	0.348
Max of Angular Acceleration-AP	34.92 ± 12.23	31.69 ± 10.40	0.191	0.194
Min of Angular Acceleration-AP	37.71 ± 13.18	32.41 ± 11.37	**0.048**	0.086
Range of Angular Acceleration-AP	72.63 ± 20.67	64.10 ± 18.17	**0.044**	0.082
RMS-Acceleration-R	0.61 ± 0.17	0.54 ± 0.16	**0.039**	**0.036**
RMS-Angular Velocity-R	3.17 ± 0.60	2.78 ± 0.58	**0.003**	**0.005**
RMS of Jerk-R	13.68 ± 4.69	12.00 ± 4.40	0.086	0.055
Max of Jerk-R	46.68 ± 20.00	38.46 ± 14.45	**0.034**	**0.012**
Min of Jerk-R	37.21 ± 14.13	30.98 ± 9.87	**0.022**	**0.028**
Range of Jerk-R	83.90 ± 31.67	69.43 ± 20.79	**0.016**	**0.008**
RMS of Angular Acceleration-R	29.91 ± 8.24	26.69 ± 11.01	0.111	**0.005**
Max of Angular Acceleration-R	61.08 ± 16.44	50.40 ± 20.55	**0.007**	**0.003**
Min of Angular Acceleration-R	82.62 ± 24.03	69.23 ± 25.73	**0.012**	**0.002**
Range of Angular Acceleration-R	143.70 ± 33.86	119.63 ± 42.25	**0.003**	**0.000**
**Turning Characteristics from Outer Turning Ankle**				
Number of Transitions/Steps	3.10 ± 1.53	2.81 ± 1.52	0.372	0.295
Angle of Turn-Average	140.06 ± 40.00	137.70 ± 49.96	0.802	0.885
Angle of Turn-Variability	54.87 ± 29.61	48.12 ± 30.10	0.288	0.348
Duration of Turn-Average	0.50 ± 0.13	0.51 ± 0.17	0.802	0.811
Duration of Turn-Variability	0.16 ± 0.11	0.17 ± 0.13	0.893	0.903
Angular Velocity-Average	429.60 ± 257.77	462.34 ± 330.44	0.594	0.745
Angular Velocity-Variability	316.77 ± 379.81	387.75 ± 506.03	0.442	0.774
Peak Angular Velocity-Average	15,597.38 ± 9,765.84	15,511.74 ± 12,573.28	0.971	0.781
Peak Angular Velocity-Variability	13,735.79 ± 7,822.24	11,264.82 ± 9,263.51	0.169	0.178
RMS-Acceleration-ML	0.48 ± 0.11	0.45 ± 0.15	0.306	0.332
RMS-Angular Velocity-ML	1.03 ± 0.35	0.91 ± 0.45	0.171	0.052
RMS of Jerk-ML	21.24 ± 6.58	17.68 ± 7.00	**0.015**	**0.041**
Max of Jerk-ML	60.78 ± 23.21	48.96 ± 19.41	**0.012**	**0.025**
Min of Jerk-ML	57.90 ± 19.34	48.54 ± 21.96	**0.033**	**0.031**
Range of Jerk-ML	118.68 ± 35.66	97.51 ± 37.32	**0.007**	**0.021**
RMS of Angular Acceleration-ML	13.70 ± 4.51	11.79 ± 5.07	0.061	**0.037**
Max of Angular Acceleration-ML	33.69 ± 10.66	26.95 ± 12.85	**0.007**	**0.002**
Min of Angular Acceleration-ML	24.97 ± 8.64	21.90 ± 9.30	0.107	0.082
Range of Angular Acceleration-ML	58.66 ± 17.37	48.85 ± 20.40	**0.015**	**0.007**
RMS-Acceleration-VT	0.35 ± 0.09	0.32 ± 0.11	0.253	0.373
RMS-Angular Velocity-VT	2.49 ± 0.64	2.19 ± 0.83	0.052	**0.017**
RMS of Jerk-VT	10.22 ± 3.59	8.93 ± 3.78	0.101	0.094
Max of Jerk-VT	25.86 ± 9.88	21.68 ± 9.33	**0.044**	0.058
Min of Jerk-VT	24.15 ± 11.35	19.60 ± 7.46	**0.034**	**0.029**

Range of Jerk-VT	50.01 ± 19.72	41.28 ± 15.76	**0.026**	**0.046**
RMS of Angular Acceleration-VT	41.91 ± 11.31	37.86 ± 16.54	0.164	**0.047**
Max of Angular Acceleration-VT	89.38 ± 23.28	82.53 ± 34.59	0.256	0.121
Min of Angular Acceleration-VT	98.72 ± 28.34	86.98 ± 32.11	0.067	0.074
Range of Angular Acceleration-VT	188.10 ± 46.29	169.51 ± 61.86	0.101	0.068
RMS-Acceleration-AP	0.38 ± 0.11	0.32 ± 0.12	**0.011**	**0.010**
RMS-Angular Velocity-AP	1.84 ± 0.48	1.83 ± 0.64	0.970	0.872
RMS of Jerk-AP	11.18 ± 3.69	9.32 ± 3.11	**0.013**	**0.037**
Max of Jerk-AP	27.39 ± 9.92	22.29 ± 7.25	**0.008**	**0.025**
Min of Jerk-AP	31.91 ± 12.99	25.04 ± 10.72	**0.009**	**0.006**
Range of Jerk-AP	59.30 ± 21.82	47.32 ± 16.62	**0.006**	**0.007**
RMS of Angular Acceleration-AP	23.17 ± 6.51	21.90 ± 8.01	0.403	0.602
Max of Angular Acceleration-AP	48.06 ± 16.57	42.97 ± 16.37	0.149	0.222
Min of Angular Acceleration-AP	44.60 ± 11.40	39.85 ± 16.66	0.106	0.157
Range of Angular Acceleration-AP	92.66 ± 23.80	82.81 ± 29.20	0.078	0.148
RMS-Acceleration-R	0.74 ± 0.16	0.66 ± 0.20	0.058	0.127
RMS-Angular Velocity-R	3.48 ± 0.64	3.22 ± 0.85	0.093	0.131
RMS of Jerk-R	17.95 ± 5.46	15.21 ± 5.18	**0.018**	0.061
Max of Jerk-R	56.67 ± 20.17	42.94 ± 15.48	**0.001**	**0.002**
Min of Jerk-R	50.11 ± 20.09	45.07 ± 19.22	0.232	0.336
Range of Jerk-R	106.77 ± 34.74	88.01 ± 30.66	**0.009**	**0.039**
RMS of Angular Acceleration-R	36.19 ± 8.94	33.35 ± 13.04	0.215	0.148
Max of Angular Acceleration-R	66.38 ± 20.38	56.74 ± 22.76	**0.036**	**0.027**
Min of Angular Acceleration-R	94.31 ± 25.52	86.80 ± 31.34	0.209	0.274
Range of Angular Acceleration-R	160.68 ± 40.54	143.54 ± 50.19	0.073	0.068

Table S2. Partial least square discriminant analysis (PLS-DA) model parameters trained on turning characteristics.

Model Trained On	Spatiotemporal Characteristics				Signal-based Characteristics				Combined Characteristics			
	Components	Q^2	R^2Y	R^2X	Components	Q^2	R^2Y	R^2X	Components	Q^2	R^2Y	R^2X
Head (HD)	5	0.074	0.429	0.757	2	0.069	0.309	0.476	2	0.127	0.374	0.416
Neck (C7)	3	0.089	0.379	0.535	2	0.107	0.348	0.473	4	0.033	0.532	0.558
Lower back (L5)	4	0.008	0.398	0.595	3	0.077	0.393	0.551	3	0.166	0.428	0.501
HD + C7	5	0.054	0.576	0.580	2	0.119	0.424	0.415	2	0.204	0.463	0.374
HD + L5	5	0.115	0.541	0.477	2	0.134	0.470	0.378	2	0.219	0.486	0.343
C7 + L5	3	0.105	0.491	0.383	2	0.137	0.436	0.415	3	0.154	0.555	0.426
Upper Body	4	0.212	0.620	0.445	2	0.134	0.505	0.380	3	0.059	0.670	0.378
Inner Turning Ankle	1	–0.009	0.071	0.225	3	0.065	0.351	0.592	3	0.033	0.385	0.528
Outer Turning Ankle	1	–0.231	0.072	0.102	1	0.031	0.081	0.433	1	0.023	0.086	0.363
Lower Body	1	–0.089	0.099	0.115	1	0.050	0.120	0.337	1	0.051	0.133	0.282
HD + Inner	2	0.154	0.382	0.304	2	0.070	0.418	0.361	4	0.013	0.665	0.452
HD + Outer	2	0.109	0.375	0.270	1	0.053	0.145	0.302	1	0.066	0.176	0.254
HD + Lower Body	2	0.050	0.424	0.205	1	0.070	0.157	0.282	2	0.051	0.470	0.294
C7 + Inner	2	0.047	0.395	0.247	4	0.002	0.590	0.519	4	0.065	0.641	0.466
C7 + Outer	1	0.165	0.279	0.143	1	0.060	0.126	0.328	1	0.075	0.154	0.281
C7 + Lower Body	1	0.157	0.308	0.107	1	0.070	0.143	0.297	2	0.038	0.487	0.298
L5 + Inner	3	0.065	0.417	0.400	3	0.023	0.499	0.435	4	0.026	0.626	0.456
L5 + Outer	3	0.113	0.453	0.379	1	0.091	0.162	0.304	2	0.079	0.471	0.311
L5 + Lower Body	2	0.153	0.425	0.225	1	0.095	0.168	0.286	2	0.108	0.445	0.301
HD + C7 + Inner	4	0.094	0.583	0.432	2	0.102	0.491	0.344	3	0.044	0.695	0.337
HD + C7 + Outer	3	0.078	0.563	0.342	1	0.063	0.150	0.303	2	0.071	0.510	0.307
HD + C7 + Lower Body	3	0.014	0.594	0.274	1	0.076	0.161	0.281	2	0.123	0.565	0.281
HD + L5 + Inner	3	0.119	0.528	0.319	2	0.119	0.496	0.330	3	0.089	0.652	0.341
HD + L5 + Outer	3	0.034	0.594	0.275	1	0.088	0.181	0.280	2	0.090	0.529	0.288
HD + L5 + Lower Body	2	0.163	0.504	0.199	2	0.006	0.512	0.307	2	0.128	0.535	0.276
C7 + L5 + Inner	3	0.055	0.505	0.324	2	0.113	0.474	0.352	4	0.001	0.713	0.415
C7 + L5 + Outer	3	0.031	0.551	0.317	1	0.090	0.158	0.307	2	0.123	0.541	0.308
C7 + L5 + Lower Body	2	0.137	0.474	0.213	1	0.095	0.166	0.286	3	0.036	0.671	0.332
Upper Body + Inner	4	0.065	0.628	0.379	2	0.131	0.535	0.336	3	0.118	0.719	0.334
Upper Body + Outer	4	0.066	0.696	0.372	2	0.013	0.527	0.333	2	0.148	0.563	0.300
Full Body	3	0.059	0.648	0.257	2	0.027	0.555	0.311	2	0.180	0.587	0.279

Table S3: Demographic characteristics of the subjects in the algorithm development set

Demographics	CL (n = 16)	PD (n = 10)	p
Age (years)	70.03 ± 8.15	73.04 ± 9.74	0.261
Height (m)	1.74 ± 0.08	1.67 ± 0.08	0.052
Mass (Kg)	83.99 ± 11.71	72.61 ± 10.41	0.048
BMI (kg/m²)	27.70 ± 4.22	26.29 ± 4.25	0.682
ABCs (0–100)%	89.77 ± 11.50	73.36 ± 23.32	0.030
MMSE (0–30)	28.60 ± 2.10	28.70 ± 1.83	0.994
LEDD, mg/day		587.70 ± 365.08	
Hoehn and Yahr (n)		HY I: 8	
		HY III: 2	
MDS-UPDRS III		41.10 ± 9.89	

ACRONYMS: BMI, Body mass index; MMSE, Mini-Mental State Examination; ABCs, Activity balance score; LEDD, Levodopa equivalent medical dosage; MDS-UPDRS, Movement disorders-Unified Parkinson's disease rating scale.
